# Affordability of current, and healthy, more equitable, sustainable diets by area of socioeconomic disadvantage and remoteness in Queensland: insights into food choice

**DOI:** 10.1186/s12939-021-01481-8

**Published:** 2021-06-30

**Authors:** Amanda Lee, Dori Patay, Lisa-Maree Herron, Ella Parnell Harrison, Meron Lewis

**Affiliations:** School of Public Health, Faculty of Medicine, University of Queensland, 266 Herston Rd, Herston, QLD 4006 Australia

**Keywords:** Food prices, Diet prices, Diet affordability, Healthy diet, Equitable diet, Sustainable diet, Socioeconomic disadvantage, Remote, INFORMAS

## Abstract

**Background:**

Poor diet is the leading preventable risk factor contributing to the burden of disease globally and in Australia, and is inequitably distributed. As the price of healthy foods is a perceived barrier to improved diets, evidence on the cost and affordability of current (unhealthy) and recommended (healthy, more equitable and sustainable) diets is required to support policy action.

**Methods:**

This study applied the Healthy Diets ASAP (Australian Standardised Affordability and Pricing) methods protocol to measure the cost, cost differential and affordability of current and recommended diets for a reference household in Queensland, Australia. Food prices were collected in 18 randomly selected locations stratified by area of socioeconomic disadvantage and remoteness. Diet affordability was calculated for three income categories.

**Results:**

Surprisingly, recommended diets would cost 20% less than the current diet in Queensland as a whole. Households spent around 60% of their food budget on discretionary choices (that is, those not required for health that are high in saturated fat, added sugar, salt and/or alcohol). Queensland families would need to spend around 23% of their income on recommended diets. However, recommended diets would not be affordable in low socioeconomic or very remote areas, costing 30 and 35% of median household income respectively. The government supplements due to the SARS-CoV-2 pandemic would improve affordability of recommended diets by 29%.

**Conclusions:**

Study findings highlight that while price is one factor affecting consumer food choice, other drivers such as taste, convenience, advertising and availability are important. Nevertheless, the study found that recommended diets would be unaffordable in very remote areas, and that low-income families are likely experiencing food stress, irrespective of where they live in Queensland. Policy actions, such as increasing to 20% the current 10% tax differential between basic healthy, and unhealthy foods in Australia, and supplementing incomes of vulnerable households, especially in remote areas, are recommended to help improve diet equity and sustainability, and health and wellbeing for all.

**Supplementary Information:**

The online version contains supplementary material available at 10.1186/s12939-021-01481-8.

## Background

Poor diet is now the leading preventable risk factor contributing to the burden of disease, globally, and in Australia [1, 2]. Poor diet is driven by food environments that encourage overconsumption of unhealthy options [3, 4]. Hence there is growing need to understand drivers of food choices and support policy action that will improve food environments to shift population diets towards dietary recommendations.

Food price and affordability are significant contributors to food security and dietary choice [5, 6]. Better information about the cost and affordability of habitual and recommended diets is required to inform potential health and fiscal policy action, such as taxes and subsidies, to manipulate food pricing to promote healthier options [5, 7–9]. To support comprehensive monitoring of food environments, the International Network on Food and Obesity/Noncommunicable disease (NCD) Research, Monitoring and Action Support (INFORMAS) has developed a step-wise framework to determine the cost and affordability of “current” diets (based on reported intake in national surveys) and “recommended” diets (consistent with dietary guidelines) [5].

This study assessed the cost, cost differential and affordability of current (unhealthy) and recommended (healthy, more equitable and sustainable) diets in the state of Queensland, Australia, by area of socioeconomic disadvantage and by remoteness. Given 67% of Australian adults and 25% of children aged two to 17 years are overweight or obese [10], and the high rates of poor-diet related outcomes in Australia [1], current diets are considered unhealthy. In Queensland, the prevalence of diet-related NCD is highest in remote locations and areas of socioeconomic disadvantage [11]. The recommended diet is consistent with the Australian Dietary Guidelines 2013 (ADGs) [12]. Contrary to recent claims [13], the recommended diet is more sustainable than the current Australian diet, being produced by food systems that use less water, support biodiversity and generate 25% less greenhouse emissions [14].

More evidence also is needed to better understand the relationships between household income and food choice [15–17]. Hence, this study also assessed, opportunistically, the impact on diet affordability of income supplements introduced by the Australian Government during the SARS-CoV-2 pandemic in 2020 [18].

## Methods

The aim of this study was to assess the cost, cost differential and affordability of current (unhealthy) and recommended (healthy, more equitable and sustainable) diets in the state of Queensland, Australia, by area of socioeconomic disadvantage and by remoteness. To achieve this, the Healthy Diets ASAP (Australian Standardised Affordability and Pricing) methods protocol [19] was applied. This protocol was developed to address the limitations of earlier approaches assessing food cost and affordability [9, 20]. The protocol is consistent with the INFORMAS framework’s ‘optimal’ approach to assess food price and affordability [5]. The background, description, collaborative development process, application and testing of the protocol have been detailed elsewhere [8, 19]. Therefore, this paper offers contextualisation and a brief explanation of methods. The Healthy Diets ASAP protocol consists of five parts: standardised current and recommended diet pricing tools; store location and sampling; calculation of median gross and indicative low disposable income; food price data collection; and analysis and reporting [19].

### Diet pricing tools

The current and recommended diet pricing tools contain the type and quantity of foods and drinks for the members of a reference household per fortnight based on intake reported in the most recent national nutrition survey data [21], and as recommended by the ADGs [12], respectively. The quantities of food per fortnight were calculated for a reference household of four: an adult male 31–50 years old, an adult female 31–50 years old, a 14 year old boy and an 8 year old girl [19]. The contents of the current and recommended diets are summarised in Table 1, detailed in Additional file 1, and illustrated pictorially elsewhere [22]. The current diet includes some healthy food and drinks, but also “discretionary” choices. Discretionary food and drinks are defined as those that are not a necessary part of the recommended diet and are high in saturated fat, added sugars, salt and/or alcohol [12]. The recommended diet comprises the healthy food and drinks commonly consumed in the current diet in optimal quantities. The diets are similar in energy content: for the reference household the current diet provides 33,860 kJ per day and the recommended diet provides 33,610 kJ per day.

**Table 1:** Foods and drinks included in the Healthy Diets ASAP diet pricing tools [19]

Current diet	Recommended diet
**• Healthy foods and drinks** as per the seven food groups on the right; in reduced amounts reflecting reported intakes (ABS, 2013) **• Artificially sweetened beverages** **• Discretionary (unhealthy) foods and drinks:** **◦ Drinks**: sugar sweetened beverages **◦ Cereals, snacks and desserts:** muffin, sweet biscuits, savoury crackers, confectionary, chocolate, potato crisps, muesli bar, mixed nuts (salted), ice cream, fruit salad (canned in juice) **◦ Processed meats:** beef sausages, ham **◦ Spreads, sauces, condiments and ingredients:** butter, tomato sauce, salad dressing, white sugar **◦ Convenience meals:** frozen lasagne, chicken soup (canned), frozen fish fillet (crumbed), instant noodles, meat and vegetable stew (canned) **◦ Fast food:** pizza, meat pie, hamburger, potato chips/fries **◦ Alcohol:** beer (full strength), white wine (sparkling), red wine, whisky	**• Water** (bottled) **• Fruit**: apples, bananas, oranges **• Vegetables:** potatoes, broccoli, white cabbage, iceberg lettuce, onion, carrot, pumpkin, tomatoes, sweetcorn (canned), four bean mix (canned), diced tomatoes (canned), baked beans (canned), frozen mixed vegetables, frozen peas, salad vegetables in sandwich **• Grain (cereals):** wholegrain cereal biscuits (Weet-bix™), rolled oats, cornflakes, wholemeal bread, white bread, white rice, white pasta, dry water crackers, bread in sandwich **• Lean meats and alternatives:** beef mince and steak, lamb chops, cooked chicken, tuna (canned), eggs, peanuts (unsalted), meat in sandwich **• Milk, yoghurt and cheese:** cheddar cheese (full fat, reduced fat), milk (full fat, reduced fat), yoghurt (full fat plain, reduced fat flavoured) **• Unsaturated oils and spreads:** olive oil, sunflower oil, canola (margarine)

### Store location and sampling

In preparation for sampling, all Queensland locations at Statistical Area Level 2 (SA2) level were stratified by area of socioeconomic disadvantage and remoteness. SA2 areas are classified by the Australian Bureau of Statistics (ABS) as medium-sized geographical areas representing communities “that interact together socially and economically” [23]. The four Socio-Economic Indexes for Areas (SEIFA) developed by the ABS rank SA2 locations based on a variety of census data [24]. The Index of Relative Socioeconomic Disadvantage (IRSD) was selected as the basis of socioeconomic disadvantage stratification in this study, and each SA2 area was assigned a quintile according to the relative IRSD ranking. SEIFA quintile 1 comprises the most disadvantaged, and SEIFA quintile 5 the least disadvantaged SA2 locations.

Remoteness was defined by the Accessibility/Remoteness Index of Australia Plus (ARIA+) categorisation, which indicates the relative access to services in different locations [25]. Levels of remoteness are expressed as: major cities, inner regional, outer regional, remote and very remote locations. Statistical Area 1 locations are geographically smaller than SA2 locations, so each SA2 location may include areas of varying designated ARIA+ classification. To determine the most appropriate ARIA+ classification, a concordance of the Queensland SA2s areas was developed, with Census data accessed to identify the population numbers at each level of remoteness within each SA2 [26]. The level of remoteness with the largest population was then assigned to the SA2 for the purposes of this study.

Following assignment of socioeconomic disadvantage levels and remoteness levels, 18 SA2 locations (falling within SEIFA quintiles 1, 3 and 5 and ARIA+ categories major cities, outer regional and very remote) were randomly selected in accordance with the Healthy Diets ASAP protocol [19].

As per the protocol, Google Maps was used to identify the prescribed food outlets within 7 km by car from the geographical centre of each SA2 location, including one outlet of each major supermarket chain and/or independent grocer, takeaway outlets of commonly consumed ‘fast foods’, independent bakeries and liquor stores [19].

### Calculation of household incomes

In accordance with the Healthy Diets ASAP protocol [19], the median gross household income (before taxation, rent and other expenses) in each SA2 area was recorded, and an indicative low disposable household income for the reference household was calculated. The indicative low disposable household income was also calculated for a time point following the onset of the SARS-CoV-2 pandemic, in order to assess the impact of additional income for people receiving income support payments (Economic Support Payment and Coronavirus Supplement) introduced by the Australian Government between May and September 2020 [18]. Thus, this study assessed diet affordability for three categories of household income.

The median gross income for the reference household per fortnight at each SA2 location was sourced from the corresponding ABS 2016 Census Community Profile [27] and adjusted by ABS Wage Price Indices to June 2019 [28]. The indicative calculated low disposable household income, based on a set of assumptions regarding the household, employment income at minimum wage, tax payable and eligible welfare payments provided by Services Australia (The Australian Government, 2020), was determined per fortnight for the reference household as detailed in the protocol [19], as at August 2019 and May 2020. Additional file 2 contains the detailed data and calculations for the indicative low disposable household income in 2019 and with the government supplements in 2020.

### Price data collection

Seven volunteers from the Queensland Country Women’s Association Country Kitchens program [29], volunteer dietitians from Queensland Health and an Indigenous community-controlled health service, and three University of Queensland research assistants were trained in the strict application of the Healthy Diets ASAP data collection protocol, including use of the survey form (Additional file 3) to collect food and drink prices in designated stores in each included location across the state. For example, the data collection protocol outlines a procedure to follow if the stipulated brands and sizes were not available or were on price promotion [19]. Permission to collect food prices from each store was requested and obtained immediately prior to data collection. Food prices were collected between May and October 2019.

### Analysis and reporting

Two research assistants (EPH and ML) double entered, cross-checked and cleaned the data from the price data survey forms into Microsoft® Office Excel (2016) spreadsheets. As per the protocol, if a value was missing, the mean price of the item in other stores in the same SA2 location was substituted [19].

Diet costs and affordability were calculated for each SA2 location, then synthesised by area of socioeconomic disadvantage (SEIFA quintile) and remoteness (ARIA+ category). The mean total costs of the current and recommended diets, as well as the cost and proportion of the total spent on different food groups and components, were calculated for the reference household per fortnight (Tables 3, 4, and 5). The affordability of current and recommended diets was calculated for households with the three different income levels (as described above). A diet was deemed to be unaffordable if it cost more than 30% of household income [7]. If the diet cost more than 25% of disposable household income, the household was considered to be in food stress [17, 30].

## Results

### Selected locations and stores

The distribution of the randomly selected locations across SA2 areas of socioeconomic disadvantage (most, median, and least disadvantaged) and three ARIA+ categories (major cities, outer regional, very remote) is presented in Table 2. None of the SA2 areas in Queensland was classified as both least disadvantaged and very remote, so no included location reflects this combination. One very remote area was a discrete Aboriginal community. Only one SA2 area was classified as median disadvantaged and very remote. In one of the 18 selected locations, data collection was not possible as management of the major food store in the community did not grant permission for food price data to be collected. Not all store types were available in each location, particularly in outer regional and very remote locations. Therefore, this paper describes the findings based on data collected in 17 locations and 125 food outlets.

**Table 2:** Stratification of the randomly selected SA2 locations based on area of socioeconomic disadvantage and remoteness

		Socioeconomic disadvantage (SEIFA)		
		Most disadvantaged (SEIFA quintile 1)	Median disadvantaged (SEIFA quintile 3)	Least disadvantaged (SEIFA quintile 5)
**Remoteness (ARIA+)**	Major cities	3	4	3
	Outer regional	2	2	1
	Very remote	2^a^	1	0

^a^ Data collection was not possible in one of these locations

### Cost of current and recommended diets

The total costs of the current and recommended diets in Queensland, by area of socioeconomic disadvantage, and by remoteness categories, are presented respectively in Tables 3, 4, and 5. These tables also display the cost of diet components by ADG food group, cost of diet contents classified as discretionary or healthy, and the affordability of the different diets.

**Table 3:** Cost of current and recommended diets and components, and diet affordability in Queensland

	**Mean total diet and food group costs for the reference household per fortnight**			
	**Current diet**		**Recommended diet**	
**Food/food groups**	**Mean cost ± SD (A$)**	**Proportion of total cost (%)**	**Mean cost ± SD (A$)**	**Proportion of total cost (%)**
Water, bottled	$18.14 ± 3.96	2%	$18.14 ± 3.96	3%
Fruit	$56.05 ± 6.13	7%	$79.66 ± 12.64	12%
Vegetables (& legumes)	$43.73 ± 3.79	5%	$111.82 ± 8.12	17%
Grain (cereal) foods	$45.52 ± 5.60	6%	$112.80 ± 12.43	18%
Lean meats, poultry, fish, eggs, nuts, seeds & alternatives	$100.35 ± 10.62	12%	$194.82 ± 24.42	30%
Milk, yoghurt, cheese & alternatives	$50.49 ± 7.23	6%	$118.31 ± 18.57	18%
Unsaturated oils and spreads	$1.28 ± 0.17	< 1%	$8.69 ± 1.41	1%
Artificially sweetened beverages	$5.99 ± 1.22	1%	–	–
Sugar sweetened beverages	$31.96 ± 5.91	4%	–	–
Takeaway foods	$160.37 ± 33.18	20%	–	–
Alcoholic beverages	$96.56 ± 6.63	12%	–	–
All other discretionary choices	$195.71 ± 41.5	24%	–	–
**Total diet**	**$806.15 ± 99.34**	**100%**	**$644.25 ± 66.28**	**100%**
Healthy foods and drinks	$321.55 ± 29.16	40%	$644.25 ± 66.28	100%
Discretionary foods and drinks	$484.60 ± 71.93	60%	–	–
	**Income and diet affordability**			
**Income categories**	**Income (A$)**		**Current diet affordability (% of income)**	**Recommended diet affordability (% of income)**
Median gross household income^a^	$3011.55		29%	23%
Indicative low disposable household income	$2358.33		34%	27%
Indicative low disposable household income including government supplements due to the SARS-CoV-2 pandemic	$3336.02		24%	19%

^a^Mean of the median gross household income of all SA2 locations within the relevant classifications included

**Table 4:** Cost of current and recommended diets and components, and diet affordability, by area socioeconomic disadvantage

	**Least disadvantaged areas (SEIFA Quintile 5)**				**Median disadvantaged areas (SEIFA Quintile 3)**				**Most disadvantaged areas (SEIFA Quintile 1)**			
**Total diet and food group costs for the reference household per fortnight**												
**Food/food groups**	**Current diet**		**Recommended diet**		**Current diet**		**Recommended diet**		**Current diet**		**Recommended diet**	
	**Mean cost ± SD (A$)**	**Proportion of total cost (%)**	**Mean cost ± SD (A$)**	**Proportion of total cost (%)**	**Mean cost ± SD (A$)**	**Proportion of total cost (%)**	**Mean cost ± SD (A$)**	**Proportion of total cost (%)**	**Mean cost ± SD (A$)**	**Proportion of total cost (%)**	**Mean cost ± SD (A$)**	**Proportion of total cost (%)**
Water, bottled	$19.84 ± 0.51	3%	$19.84 ± 0.51	3%	$19.07 ± 1.59	2%	$19.07 ± 1.59	3%	$15.91 ± 5.77	2%	$15.91 ± 5.77	2%
Fruit	$54.73 ± 5.12	7%	$76.03 ± 10.72	12%	$56.45 ± 7.60	7%	$81.83 ± 13.71	13%	$56.48 ± 4.47	7%	$79.56 ± 11.94	12%
Vegetables (& legumes)	$42.45 ± 4.09	5%	$110.45 ± 6.33	18%	$44.81 ± 4.09	5%	$112.86 ± 10.85	18%	$43.33 ± 2.74	5%	$111.53 ± 4.50	17%
Grain (cereal) foods	$44.17 ± 4.36	6%	$108.41 ± 5.61	17%	$45.95 ± 3.76	6%	$113.79 ± 9.12	18%	$45.91 ± 7.63	6%	$114.59 ± 17.40	17%
Lean meats, poultry, fish, eggs, nuts, seeds & alternatives	$95.14 ± 4.04	12%	$185.84 ± 9.93	30%	$99.91 ± 8.21	12%	$192.21 ± 17.52	30%	$104.33 ± 13.99	13%	$203.85 ± 33.51	31%
Milk, yoghurt, cheese & alternatives	$49.41 ± 1.96	6%	$118.99 ± 8.95	19%	$48.45 ± 4.90	6%	$113.44 ± 10.97	18%	$53.60 ± 10.11	7%	$123.55 ± 26.97	19%
Unsaturated oils and spreads	$1.26 ± 0.14	< 1%	$8.21 ± 0.77	1%	$1.27 ± 0.14	< 1%	$8.85 ± 1.02	1%	$1.31 ± 0.21	< 1%	$8.83 ± 1.96	1%
Artificially sweetened beverages	$5.53 ± 14.74	1%	–	–	$5.96 ± 27.55	1%	–	–	$6.33 ± 35.95	1%	–	–
Sugar sweetened beverages	$29.89 ± 2.52	4%	–	–	$31.54 ± 3.96	4%	–	–	$33.82 ± 8.37	4%	–	–
Takeaway foods	$156.94 ± 5.36	20%	–	–	$168.80 ± 1.80	21%	–	–	$152.83 ± 9.35	19%	–	–
Alcoholic beverages	$97.30 ± 7.94	13%	–	–	$98.66 ± 66.49	12%	–	–	$93.62 ± 102.02	12%	–	–
All other discretionary choices	$181.58 ± 11.51	23%	–	–	$198.41 ± 40.49	24%	–	–	$201.97 ± 51.95	25%	–	–
**Total diet**	**$778.25 ± 136.99**	**100%**	**$627.77 ± 27.91**	**100%**	**$819.28 ± 93.16**	**100%**	**$642.04 ± 60.41**	**100%**	**$809.43 ± 130.17**	**100%**	**$657.83 ± 85.44**	**100%**
Healthy foods and drinks	$312.54 ± 14.74	40%	$627.77 ± 27.91	100%	$321.87 ± 27.55	39%	$642.04 ± 60.41	100%	$327.20 ± 35.95	40%	$657.83 ± 85.44	100%
Discretionary foods and drinks	$465.72 ± 5.46	60%	–	–	$497.41 ± 66.4	61%	–	–	$482.23 ± 95.20	60%	–	–
**Income and diet affordability**												
**Income categories**	**Income (A$)**		**Current diet affordability (% of income)**	**Recommended diet affordability (% of income)**	**Income (A$)**		**Current diet affordability (% of income)**	**Recommended diet affordability (% of income)**	**Income (A$)**		**Current diet affordability (% of income)**	**Recommended diet affordability (% of income)**
Median gross household income^a^	$4241.61		18%	15%	$3040.52		27%	21%	$2157.72		38%	30%
Indicative low disposable household income	$2358.33		33%	27%	$2358.33		35%	27%	$2358.33		34%	28%
Indicative low disposable household income including government supplements due to the SARS-CoV-2 pandemic	$3336.02		23%	19%	$3336.02		25%	19%	$3336.02		24%	20%

^a^Mean of the median gross household income of all SA2 locations within the relevant classifications included

**Table 5:** Cost of current and recommended diets and components, and diet affordability, by remoteness

	**Major Cities**				**Outer Regional**				**Very Remote**			
**Total diet and food group costs for the reference household per fortnight**												
**Food/food groups**	**Current diet**		**Recommended diet**		**Current diet**		**Recommended diet**		**Current diet**		**Recommended diet**	
	**Mean cost ± SD (A$)**	**Proportion of total cost (%)**	**Mean cost ± SD (A$)**	**Proportion of total cost (%)**	**Mean cost ± SD (A$)**	**Proportion of total cost (%)**	**Mean cost ± SD (A$)**	**Proportion of total cost (%)**	**Mean cost ± SD (A$)**	**Proportion of total cost (%)**	**Mean cost ± SD (A$)**	**Proportion of total cost (%)**
Water, bottled	$20.35 ± 1.64	3%	$20.35 ± 1.64	3%	$15.58 ± 3.76	2%	$15.58 ± 3.76	2%	$13.46 ± 4.63	1%	$13.46 ± 4.63	2%
Fruit	$53.38 ± 4.08	7%	$72.81 ± 7.97	12%	$56.14 ± 1.83	7%	$83.42 ± 5.57	13%	$69.21 ± 4.34	7%	$104.53 ± 8.62	13%
Vegetables (& legumes)	$43.59 ± 1.88	6%	$110.36 ± 5.38	18%	$41.17 ± 3.64	5%	$108.90 ± 6.54	17%	$50.82 ± 2.18	5%	$126.44 ± 7.97	16%
Grain (cereal) foods	$44.34 ± 1.98	6%	$109.99 ± 2.36	18%	$43.05 ± 4.62	6%	$106.24 ± 7.12	17%	$57.59 ± 5.11	5%	$143.29 ± 9.09	18%
Lean meats, poultry, fish, eggs, nuts, seeds & alternatives	$96.45 ± 3.63	12%	$184.52 ± 8.71	30%	$97.95 ± 5.67	13%	$192.07 ± 9.74	31%	$125.84 ± 8.47	12%	$253.20 ± 22.25	31%
Milk, yoghurt, cheese & alternatives	$47.93 ± 2.75	6%	$112.59 ± 8.35	18%	$48.96 ± 2.01	6%	$113.03 ± 3.88	18%	$67.13 ± 8.97	6%	$160.13 ± 23.70	20%
Unsaturated oils and spreads	$1.27 ± 0.06	< 1%	$8.42 ± 0.38	1%	$1.15 ± 0.12	< 1%	$7.86 ± 0.75	1%	$1.65 ± 0.10	< 1%	$12.13 ± 0.97	1%
Artificially sweetened beverages	$5.64 ± 10.21	1%	–	–	$5.64 ± 14.02	1%	–	–	$8.85 ± 12.57	1%	–	–
Sugar sweetened beverages	$31.14 ± 1.57	4%	–	–	$28.19 ± 3.11	4%	–	–	$45.43 ± 6.37	4%	–	–
Takeaway foods	$149.31 ± 6.05	19%	–	–	$156.40 ± 2.36	20%	–	–	$225.57 ± 10.16	21%	–	–
Alcoholic beverages	$96.36 ± 8.50	12%	–	–	$99.74 ± 51.60	13%	–	–	$89.62 ± 34.49	8%	–	–
All other discretionary choices	$182.42 ± 9.50	24%	–	–	$178.80 ± 15.87	23%	–	–	$304.41 ± 12.10	29%	–	–
**Total diet**	**$772.20 ± 14.18**	**100%**	**$619.04 ± 22.66**	**100%**	**$772.68 ± 66.04**	**100%**	**$627.10 ± 21.12**	**100%**	**$1059.57 ± 36.90**	**100%**	**$813.18 ± 0.00**	**100%**
Healthy foods and drinks	$312.96 ± 10.21	41%	$619.04 ± 22.66	100%	$309.54 ± 14.02	40%	$627.10 ± 21.12	100%	$394.54 ± 12.57	37%	$813.18 ± 0.00	100%
Discretionary foods and drinks	$459.24 ± 7.54	59%	–	–	$463.14 ± 49.74	60%	–	–	$665.03 ± 24.33	63%	–	–
**Income and diet affordability**												
**Income categories**	**Income (A$)**		**Current diet affordability (% of income)**	**Recommended diet affordability (% of income)**	**Income (A$)**		**Current diet affordability (% of income)**	**Recommended diet affordability (% of income)**	**Income (A$)**		**Current diet affordability (% of income)**	**Recommended diet affordability (% of income)**
Median gross household income^a^	$3188.09		24%	19%	$2900.56		27%	22%	$2406.35		44%	34%
Indicative low disposable household income	$2358.33		33%	26%	$2358.33		33%	27%	$2358.33		45%	34%
Indicative low disposable household income including government supplements due to the SARS-CoV-2 pandemic	$3336.02		23%	19%	$3336.02		23%	19%	$3336.02		32%	24%

^a^Mean of the median gross household income of all SA2 locations within the relevant classifications included

Overall, in Queensland, the mean cost of the current diet for the reference household was A$806.15 ± 99.34 per fortnight, which was 20% more expensive than the mean cost of the recommended diet at A$644.25 ± 66.28 per fortnight (Table 3). The current diet was more expensive than the recommended diet in all surveyed locations, regardless of the level of socioeconomic disadvantage or remoteness (Tables 4 and 5, Additional file 4).

In all locations, discretionary foods and drinks comprised approximately 60% of the current diet cost for the reference household and healthy food and drinks the remaining 40% (Table 3). Takeaway foods comprised 20%, alcoholic drinks 12%, and sugar sweetened beverages (SSBs) 4% of the current diet cost (Table 3).

**Fig. 1:**
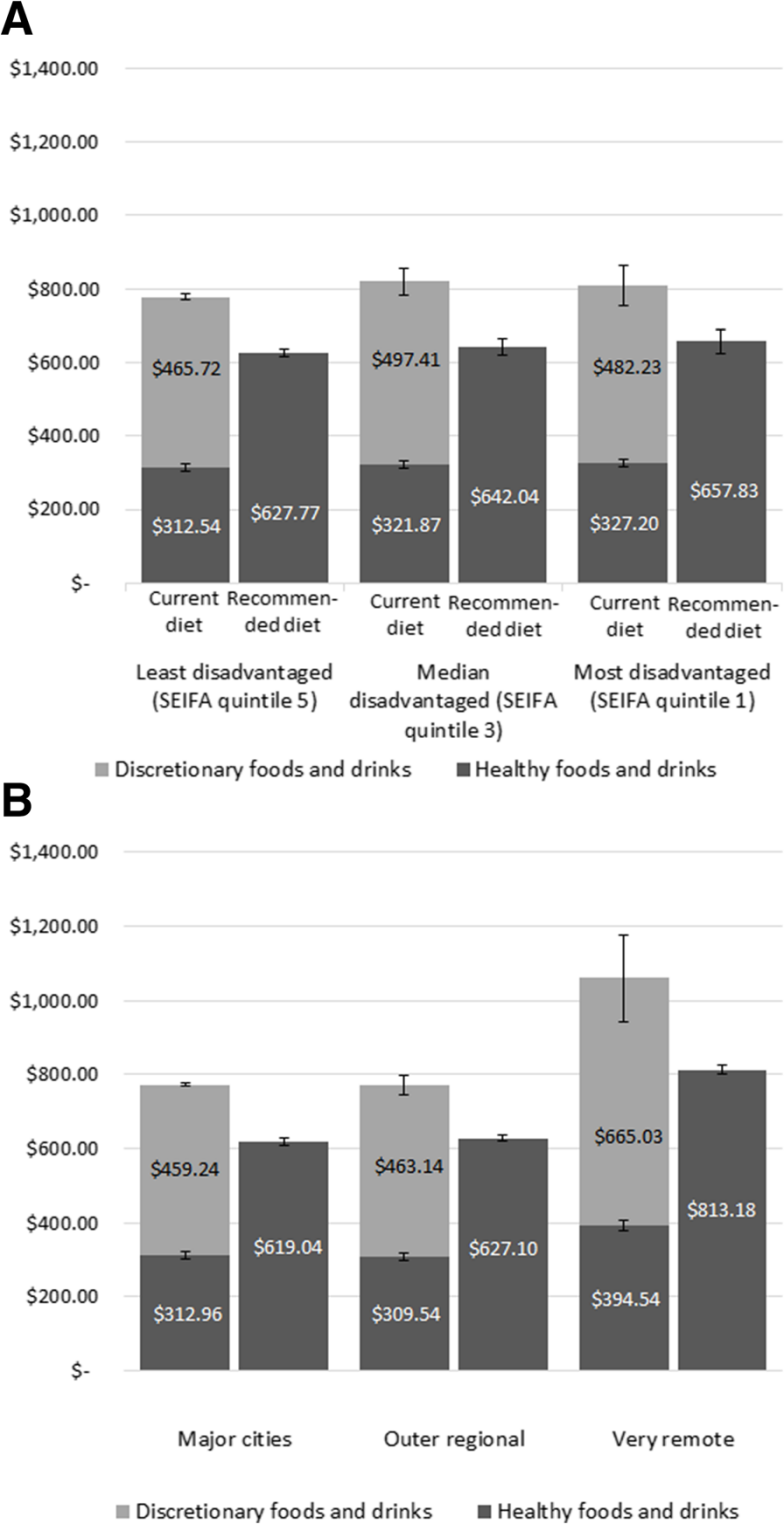
**A** Cost of current and recommended diets for reference household per fortnight by area socioeconomic disadvantage. **B** Costs of current and recommended diets for the reference household per fortnight by remoteness

**Fig. 2:**
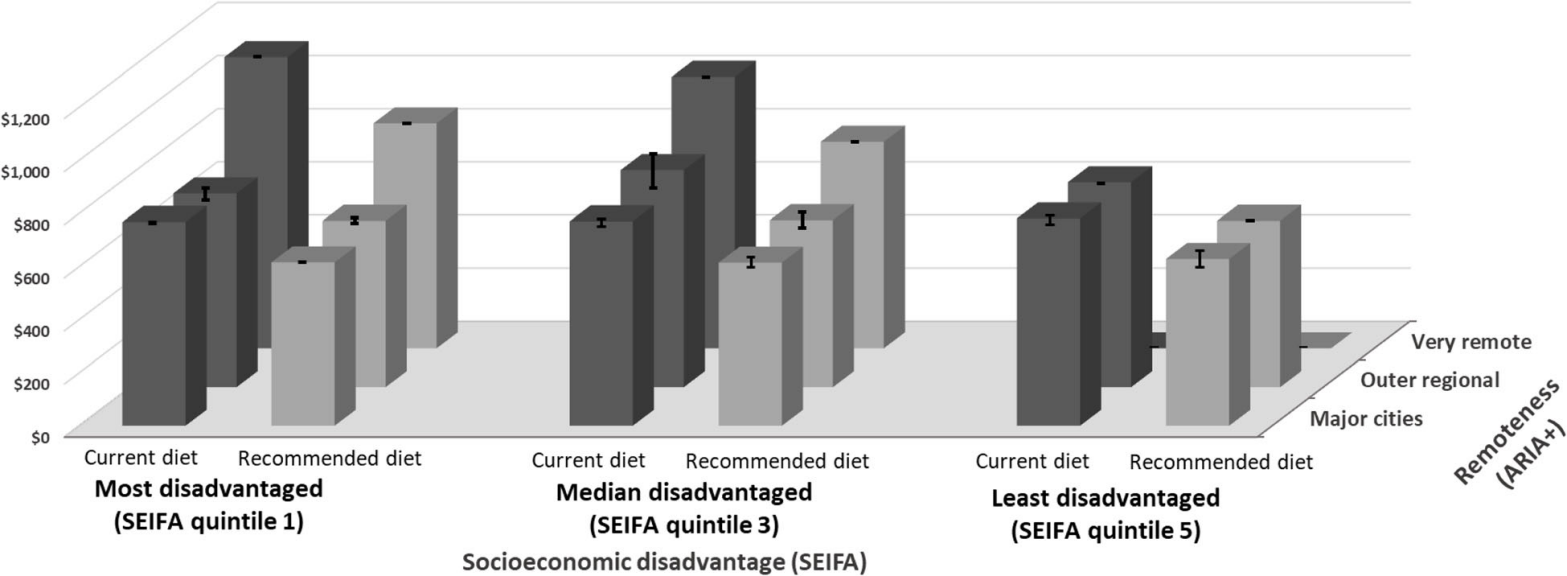
Cost of current and recommended diets per fortnight by area socioeconomic disadvantage and remoteness Error bars indicate the standard error

Figure 1A and B summarise the mean and component costs of the current and recommended diets by area of socioeconomic disadvantage and remoteness categories respectively. The costs of the current and recommended diets by area of socioeconomic disadvantage and remoteness combined are shown in Fig. 2.

The current diet was most expensive per fortnight in the median disadvantaged areas (A$819.28 ± 93.16), followed by the most disadvantaged areas (A$809.43 ± 130.17), and least expensive in the least disadvantaged locations (A$778.25 ± 136.99). However, the cost of the recommended diet would increase by 5% linearly from the least disadvantaged (A$627.77 ± 27.91 per fortnight) to the most disadvantaged areas (A$657.83 ± 85.44 per fortnight) (Fig. 1A, Table 4). The cost differential between the current and recommended diet was highest (22%) in the median disadvantaged locations, and 19% in both the least and most disadvantaged areas.

The costs of the current and recommended diets were similar in major cities and outer regional areas; however, the costs of both were much higher (37 and 31% respectively) in very remote areas in Queensland (Fig. 1B, Table 5). The highest cost differential between the current and recommended diet was 23% in the very remote areas. The differential was 20% in major cities and 19% in outer regional locations.

The proportion spent on discretionary items was highest in very remote areas at 63% (A$665.03 ± 24.33 per fortnight) of expenditure on food and drinks in the current diet (Fig. 1B, Table 5).

The dominant effect of remoteness on diet cost is evident in Fig. 2. In the most socioeconomically disadvantaged areas, the costs of current and recommended diets per fortnight were 43% (A$329.22) and 38% (A$232.36) higher respectively in very remote locations than in major cities in Queensland.

### Affordability of current and recommended diets

**Fig. 3:**
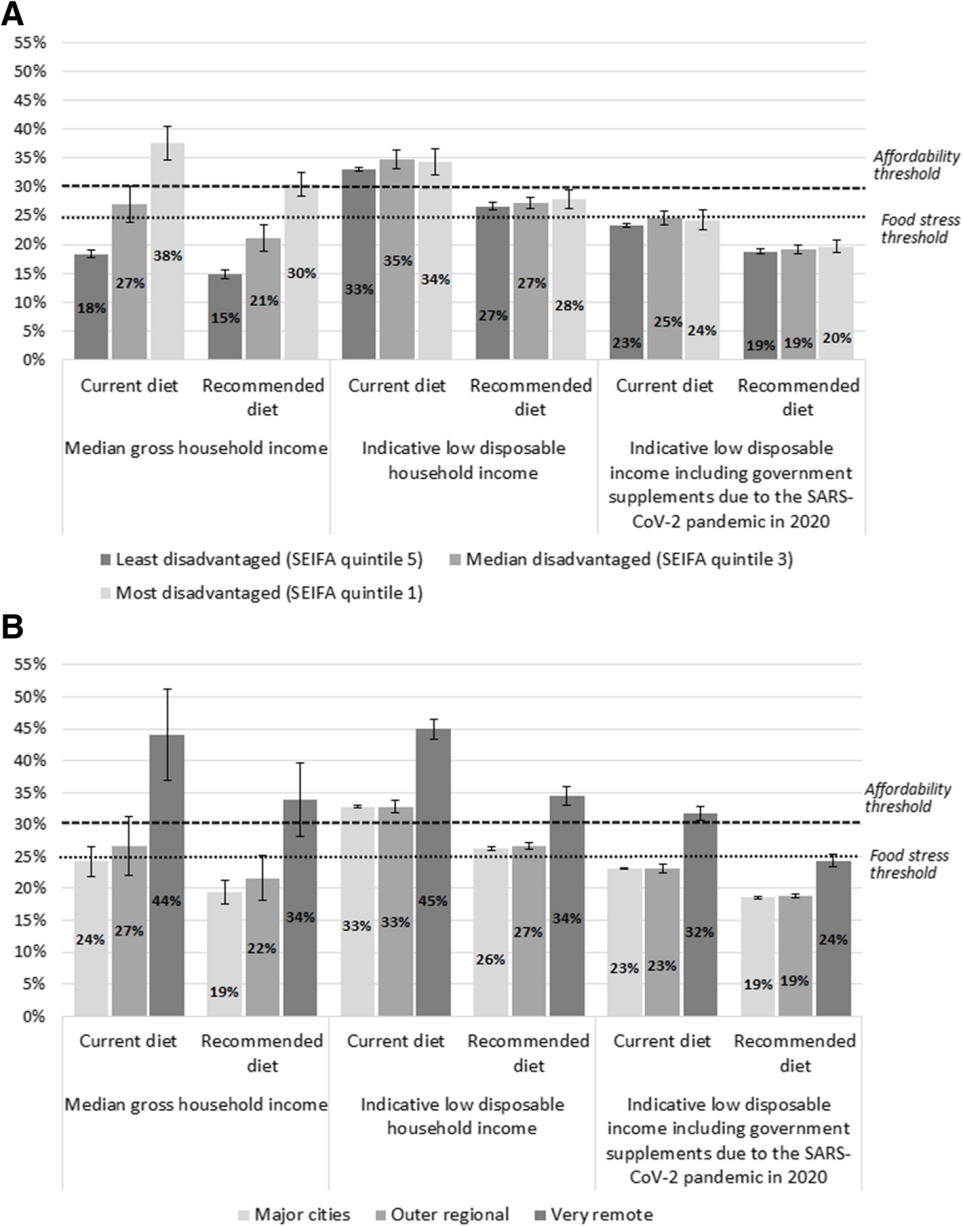
**A** Affordability of current and recommended diets, for reference household, by income and area socioeconomic disadvantage. **B** Affordability of current and recommended diets, for reference household, by income and remoteness

The affordability of current and recommended diets in Queensland – calculated means, and analyses by area of socioeconomic disadvantage and by remoteness categories – is detailed in Tables 3, 4, and 5. Results for the latter two analyses are summarised in Fig. 3A and B.

On average in Queensland (mean diet affordability across the sample SA2 locations), 29 and 23% of the median gross household income was required to purchase the current diet and recommended diet respectively (Table 3). For low-income households in Queensland as a whole, the current diet was not affordable at 34% of disposable income, and the proportion of household income required to be spent on a recommended diet (27%) would exceed the food stress threshold (Table 3). The indicative low disposable household income in Queensland (the same in each location) was A$2358.33 per fortnight in June 2019. With the government supplements due to the SARS-CoV-2 pandemic this increased to A$3336.02 per fortnight between May and September 2020, increasing affordability of recommended diets by 29% (Table 3).

The current diet was unaffordable for households with low incomes in all socioeconomic areas assessed, costing 33–35% of disposable income (Fig. 3A, Table 4). Affordability of the recommended diet (27–28%) would exceed the food stress threshold for low-income families in all socioeconomic areas assessed (Fig. 3A, Table 4).

In the most disadvantaged areas, the current and recommended diets were unaffordable at 38 and 30% of the median gross household income respectively (Fig. 3A, Table 4). In the least disadvantaged areas, both the current and recommended diets were much more affordable, at 18 and 15% of median gross household income respectively (Fig. 3A, Table 4). Surprisingly, the median gross household income was less than the indicative low disposable household income in the areas of most socioeconomic disadvantage (Table 4).

Both current and recommended diets were least affordable for families living in very remote areas in Queensland (Fig. 3B, Table 5). In very remote areas, the current diet cost 44% of total income of families with median gross household income; this proportion would be 34% for the recommended diet (Fig. 3B, Table 5). In contrast, in major cities the current diet cost 24%, and the recommended diet cost 19%, of median gross household income (Fig. 3B, Table 5).

In very remote areas, both diets were even less affordable for families with the indicative low disposable household income: the current diet would cost 45% of their income, and the recommended diet would cost 34% (Fig. 3B, Table 5). In major cities, the current diet cost 33%, while the recommended diet would cost 26%, of the disposable income of low-income households (Fig. 3B, Table 5).

Under the welfare policy settings in place from May 2020 to September 2020 in response to the SARS-CoV-2 pandemic, recommended diets would be more affordable for Queensland households eligible for additional benefits than they were before the pandemic (Table 3). The proportion of disposable household income needed for the reference household to purchase a recommended diet reduced from 27 to 19% (Table 3), improving its affordability by around 29%, with similar findings in all areas of socioeconomic disadvantage (Table 4, Fig. 3A) and remoteness (Table 5, Fig. 3B).

## Discussion

### Methodological considerations

For the first time, this study applied the Healthy Diets ASAP methods protocol to assess and compare the cost, cost differentials and affordability of current and recommended diets across locations stratified by area of socioeconomic disadvantage and remoteness in the state of Queensland, Australia. Previously there had been no such systematic assessment of cost and affordability of diets using standardised methods throughout the state. Hence, these are the first results that can be compared to other survey findings and used to inform potential policy responses to help improve population diet.

Queensland was one of the first jurisdictions in Australia to report regularly on food prices from a public health perspective, through Healthy Food Access Basket surveys [31, 32]. However, the ‘healthy basket’ diet pricing tool used until 2014 did not align with the ADGs as, while the diet was healthier than usual diets, it included items that were not recommended by the ADGs at the time, such as sugar, sausages, cake, and chocolate [20]. Further, the cost of the current diet was not assessed for comparison, and determination of household income lacked rigour and consistency [20]. Methodological differences in other jurisdictions made comparison of results difficult; these included variations in the composition of reference households, the included food items, calculation of household income, protocol of store sampling, and data collection [20].

### Diet costs

The finding that recommended diets would be 20% less expensive than current diets in all surveyed locations in Queensland aligns with results of other studies in Australia that have used the Healthy Diets ASAP methods – in capital cities Canberra and Sydney [33] and Brisbane [8, 19], as well as in country Victoria [34] and remote Aboriginal communities [35] and, using streamlined methods, nationally [36]. These studies confirm that current diets cost 14 to 23% more than recommended diets under current Australian policy settings, which include the exemption of basic healthy foods from a 10% Goods and Service Tax (GST) [37]. Results are also consistent with those of a similar study in New Zealand [38], and related studies in the Northern Territory of Australia [39, 40], and in one supermarket chain in Australia [13]. However, the findings contradict the result of an earlier study conducted in Greater Western Sydney, which included fewer food items and focused on sustainability [15]. A previous international systematic review and meta-analysis (which did not include Australian studies) [41], also found that recommended diets were more expensive than less healthy options – although only by US$1.48/day. As with Barosh and colleagues’ study [15], most studies included in the review did not include alcohol in the current diet, potentially missing substantial costs (12% of the total diet cost in the current study, for example). Many food and diet pricing studies, including a recent Belgian study assessing costs of diets based on proportion of ultra-processed foods, measure costs per kcal or kJ, which can be methodologically spurious, conflating energy content and (high) energy density of unhealthy foods [42]. Such studies confirm the need for standardised diet pricing methods [5, 9, 20].

Other studies using the Healthy Diets ASAP method have found that food prices tend to be highest in the least socioeconomically disadvantaged areas [19, 33]. However, this study has shown that, due to the confounding effect of remoteness, the recommended diet would be most expensive in the most disadvantaged locations. Diet costs increased markedly with remoteness, with total diet costs being 34% higher in very remote areas than in major cities. These results confirm the general findings of other Australian studies [20], including in Queensland [32, 43], Western Australia [44], the Northern Territory [39, 45] and South Australia [17]. A recent Inquiry into food pricing and food security in remote Indigenous communities by the Australian Parliament’s Standing Committee on Indigenous Affairs [46] found that the relatively high cost of food and drinks in very remote areas is the consequence of high freight, operational and maintenance costs; logistical challenges; and small populations of consumers, which limit stores’ buying power. Other studies also suggest these higher prices are explained by the low density of food outlets in very remote communities, resulting in lack of competition [47]. However, amenability of intervention in remote community stores, particularly those in discrete Aboriginal communities, is exemplified by implementation of store nutrition pricing policies, including cross-subsidisation of the price of healthy foods and drinks [48], which helps explain why the greatest cost differential between the current and recommended diets in this study (23%) occurred in very remote areas.

### Drivers of food choice

The finding that a reference family of two adults and two children spent 60% of their food budget on discretionary foods and drinks is similar to the results of previous studies using the Healthy Diets ASAP approach, which found discretionary items comprised around 58% of the current diet cost [8, 33]. It is also consistent with the ADG Price Index [49], which used household expenditure data and showed discretionary items accounted for 58.2% of Australians’ spending on food and drinks. The relative proportions of the food and drink budget that households spent on alcohol (12%), takeaway meals (20%), and SSBs (4%) are similar to the proportions identified in previous studies in Sydney and Canberra [33].

This study showed that transitioning from current to recommended diets would save Queensland families on average A$161.90 per fortnight, yet the current diet reflects what the majority of Australians actually report eating. So why do people persist with their current diet if it is more expensive than a healthy, more equitable and sustainable diet? Although multiple scholars have argued that price is an important factor in consumer food choice [5, 50], this study’s findings highlight that it is not the only key determinant.

There is surprisingly little comprehensive research on the most influential drivers of consumer food choices. Multiple studies have cited the importance of particular determinants, such as taste [51, 52], healthiness/nutrition [53], time and convenience [54], gender [55], psychological or behavioural factors [54], societal influence [51], accessibility [6], packaging and labelling [56, 57], advertising, marketing, and promotion [55, 58], availability [6, 54, 59], and sociocultural acceptability [54]. Food store practices, including placement of healthy and unhealthy products, the amount of shelf-space allocated to these [60], and promotions and pricing policies, such as subsidising fresh foods [48, 61] have been cited as important factors in consumer food choice too. Some scholars consider that the *perception* that a recommended diet costs more than current diets is a contributing factor to food choice, especially in low-income households [54].

Choice experiments aiming to rank drivers of food choice have returned conflicting results; however, these have been conducted in different population groups, from various countries, cultures, age groups, educational opportunities and settings. Key factors identified included taste, nutrition, cost, and convenience [53, 62–64]. However, the validity of the data in many self-reported studies is questionable, as social desirability may have influenced the participants’ responses.

Growing evidence highlights the role of unhealthy commodity industries in driving dietary behaviours and food choice, through well-tested market and non-market activities that aim to increase the affordability, availability, accessibility, and acceptability of discretionary foods and drinks [65]. The market activities of unhealthy commodity industries include building demand through advertising, marketing, and promotion practices, and by market expansion via trade and investment liberalisation [66]. Non-market activities comprising corporate social and political activities aim to create a positive perception of the industry in the eyes of the public and policy makers, positioning industries to shape regulatory environments to benefit their profits [67]. The growing economic power of these industries enhances their abilities to pursue such activities [65]. As this study confirms, Australian households buying the current diet spent the bulk (60%) of their food budget on discretionary foods and drinks. Hence commercial industries that produce, distribute and market these foods benefit from the status quo, and may actively seek to undermine efforts to improve diets [19, 33, 68].

### Diet affordability and food security

This study demonstrates clearly that affordability of recommended diets is influenced by both food prices and household income. Hence low-income families, especially those living in very remote areas where food prices are highest and household incomes are lowest, are most vulnerable to poor diets in Australia. The study found that recommended diets are unaffordable in very remote areas, but also showed that low-income families are likely to be experiencing food stress irrespective of where they live in Queensland. This is an important finding given the established association between low socioeconomic factors and suboptimal diets [12, 69, 70].

Diet affordability is a key component of food security. Food security is “when all people, at all times, have physical and economic access to sufficient, safe and nutritious food to meet their dietary needs and food preferences for an active and healthy life” [71]. In Australia, when assessed against a lesser definition (living in a household that ran out of food in the last 12 months and couldn’t afford to buy more) in the National Nutrition and Physical Activity Survey (NNPAS) 2011–13 [21], 4% of Australians experienced food insecurity. When more sensitive methods were used, 36% of Australians were found to experience low food security [72]. Another more recent study using the same methods as the NNPAS (2011–13) found 80% of Australian households receiving welfare support experienced food insecurity [73]. Such results highlight the inadequacy of welfare payments, which need to be increased [74]. This study demonstrates that the government supplements introduced due to the SARS-CoV-2 pandemic improved the affordability of recommended diets for vulnerable families by 29%. A recent Australian survey found that following the implementation of the supplements, 83% of welfare-dependent people reported eating healthier and more regularly than they did before the pandemic [75]. These findings are consistent with the positive outcomes of evaluations of food voucher subsidy programs in the USA [76, 77].

The finding that the indicative low disposable household income was higher than the median gross household income in the areas of most socioeconomic disadvantage in Queensland may reflect that a high proportion of households in those locations rely solely on welfare income (e.g. aged pension or unemployment benefits) and/or on a single income (e.g. single parent families or single aged pensioners). These households would receive an income lower than the indicative low disposable household income used in this study. Hence, this study presents the best-case scenario for the most vulnerable households.

### Recommendations

Despite growing evidence that the recommended diet would be more affordable than the current diet in Queensland and Australia, most households consume the latter. Therefore, it is important to address environmental factors influencing dietary choices, including food prices.

The affordability of recommended diets should be protected through pricing and taxation policies, including retention of the exemption of basic healthy foods from GST in Australia. However, the results of the study suggest that the 10% tax differential may not be great enough to encourage consumption of recommended diets, consistent with evidence of the benefits of increasing taxation on specific unhealthy foods and drinks (e.g. SSBs) by 20% [78]. Therefore, it is recommended that GST on *all* unhealthy foods and drinks in Australia should be increased to 20%, as supported by previous modelling [8].

Welfare benefits should be increased permanently beyond pre-pandemic levels to at least the levels assessed in this study [70], as advocated by the Australian Council of Social Service [79].

Action on the social and commercial determinants of diet and health is required, including key policy actions on regulation of advertising, marketing, and promotion activities of unhealthy food and alcohol industries [65] and in-store nutrition policies, such as allocating more shelf space for healthy foods than discretionary items, and replacing the latter with healthy items at checkouts and end-of-aisle displays [80].

This study offers useful insights into the economic inequities in accessing healthy food and drinks in rural Australia [81], but further diet costing studies are required in remote Aboriginal communities specifically [35, 46]. Improving nutrition and health requires a genuine commitment to improving food security through reform that ensures Aboriginal and Torres Strait Islander people are the decision-makers to address the required structural and systemic changes [82].

There is a need to better investigate systematically the many factors affecting affordability, availability, accessibility, and acceptability of healthy foods, and thus better understand the drivers of food choice and barriers to food security.

In Australia, a national nutrition benchmarking, monitoring and reporting system, including diet cost and affordability, is needed urgently to better understand the risk of food insecurity including the increasing risk related to the SARS-CoV-2 pandemic [83, 84] and climate change [85].

### Limitations

The methodological limitations of the Healthy Diets ASAP approach, including the implications of focusing on mean population intake to standardise methods for comparison, have been described previously [8, 19, 33]. While the indicative low income was calculated to reflect the reference household’s disposable income, the median gross income values reflect the pre-tax income. Because of this limitation, diets may be even less affordable for families with median gross household income than suggested.

Data on food and drink prices in early and mid-2020, which could not be collected because of SARS-CoV-2-related restrictions, would have assisted further analysis of the impact of the government supplements in low-income households. However, the lack of these data does not change the applicability of key findings – increasing welfare income has the potential to significantly increase affordability of recommended diets. Lessening of restrictions enabled food price data collection in stores in August/September 2020; this report is forthcoming.

The randomly selected SA2 locations included only one very remote location in an area of most disadvantage; inclusion of more very remote locations may be necessary to better understand the role of diet cost and affordability on consumer food choices in very remote communities.

## Conclusions

This study highlighted the value of assessing the cost, cost differentials and affordability of current (unhealthy) and recommended (healthy, more equitable and sustainable) diets in the state of Queensland, Australia, by area of socioeconomic disadvantage and by remoteness. It showed that, although recommended diets can be less expensive than current diets, they are still unaffordable for low-income households, particularly those in very remote communities. The findings highlight the need for more systematic research on the drivers of food choice, especially in vulnerable groups, and inform several recommendations for policy actions to improve food environments and food security. These include: maintaining exemption of basic healthy foods from GST and increasing the rate of GST on all discretionary food and drinks to 20%; supporting the development and implementation of a regular, comprehensive, food and nutrition monitoring and surveillance program in Australia that includes assessment of the cost, cost differentials and affordability of current and recommended diets for key population sub-groups; and supplementing incomes of vulnerable households, especially in remote areas, to help improve diet equity and sustainability, and health and wellbeing for all.

## Supplementary Information

**Additional file 1.** Details of the current and recommended diets.

**Additional file 2.** Calculations of indicative low disposable household incomes.

**Additional file 3.** Healthy Diets ASAP Survey Form.

**Additional file 4.** Cost and affordability of the current and recommended diets in the sampled locations.

## Data Availability

The datasets supporting the conclusions of this article are included within the article and its following additional files.

## Abbreviations

ABS: Australian Bureau of Statistics
ADGs: Australian Dietary Guidelines
ARIA+: Accessibility/Remoteness Index of Australia Plus
GST: Goods and Services Tax
Healthy Diet ASAP methods protocol: Healthy Diets Australian Standardised Affordability and Pricing methods protocol
INFORMAS: International Network on Food and Obesity/Noncommunicable disease Research, Monitoring and Action Support
IRDS: Index of Relative Socioeconomic Disadvantage
NCD: Noncommunicable disease
SA2: Statistical Area Level 2
SEIFA: Socio-Economic Indexes for Areas
SSBs: Sugar sweetened beverages
