# Supplementary material for: Affordability of current, and healthy, more equitable, sustainable diets by area of socioeconomic disadvantage and remoteness in Queensland: insights into food choice
Source: Int J Equity Health. 2021 Jun 30;20:153. doi: 10.1186/s12939-021-01481-8 (PMC8243618; doi:10.1186/s12939-021-01481-8)
Supplement: Supplementary file 1 — Additional file 1. Details of the current and recommended diets. [file 12939_2021_1481_MOESM1_ESM.docx]

|  | **Current (unhealthy diet)** | **Recommended (healthy, more equitable and sustainable) diet** |  | **Current (unhealthy diet)** | **Recommended (healthy, more equitable and sustainable) diet** |  | **Current (unhealthy diet)** |
| --- | --- | --- | --- | --- | --- | --- | --- |
| **Total energy of basket per day per household** | **33860 kJ/day** | **33610 kj/day** | ***Grain (cereal) foods*** |  |  | ***Discretionary choices**** |  |
|  |  |  | Wholegrain cereal biscuits, Weetbix^TM^ (g) | 430 | 2216 | Beer, full strength (ml) | 4661 |
|  |  |  | Wholemeal bread, pp (g) | 1054 | 4272 | White wine, sparkling (ml) | 863 |
|  |  |  | Rolled oats, whole (g) | 870 | 6648 | Whiskey (ml) | 266 |
| **Food (per fortnight)** | | | White bread, pp (g) | 3033 | 893 | Red wine (ml) | 1078 |
| Bottled water, still (ml) | 5296 | 5296 | Cornflakes (g) | 680 | 670 | Butter (g) | 280 |
| Artificially sweetened beverages (ml) | 2391 | N/A | White pasta, spaghetti (g) | 1326 | 2042 | Muffin, commercial (g) | 1455 |
| **Core five food groups** |  |  | White rice, medium grain (g) | 1622 | 2042 | Cream-filled sweet biscuit, pp (g) | 496 |
| ***Fruit*** |  |  | Dry water cracker biscuit (g) | 259 | 781 | Muesli bar, pp (g) | 373 |
| Apples, red, loose (g) | 3497 | 5460 | Bread in sandwich (g) | 120 | 120 | Mixed nuts, salted (g) | 255 |
| Bananas, Cavendish, loose (g) | 899 | 5460 | ***Meats, poultry, fish, eggs, nuts, seeds and alternatives*** |  |  | Pizza, commercial (g) | 1182 |
| Oranges, loose (g) | 1664 | 5460 | Beef mince, lean (g) | 267 | 1168 | Savoury flavoured biscuits (g) | 222 |
| Fruit salad, canned in juice (g) | 2046 | N/A | Lamb loin chops (g) | 257 | 1169 | Confectionary (g) | 418 |
| Fruit juice (ml) | 3026 | N/A | Beef rump steak (g) | 1056 | 1172 | Chocolate (g) | 441 |
| ***Vegetables*** |  |  | Tuna, canned in vegetable oil (g) | 1052 | 1841 | Sugar sweetened beverages, Coca Cola (ml) | 12012 |
| Potato, white, loose (g) | 1460 | 2320 | Whole barbecue chicken, cooked (g) | 1661 | 1471 | Meat pie, commercial (g) | 1638 |
| Sweetcorn, canned, no added salt (g) | 206 | 1160 | Eggs (g) | 872 | 2208 | Frozen lasagne, pp (g) | 4322 |
| Broccoli, loose (g) | 422 | 1470 | Meat in tinned meat and vegetable casserole (g) | 646 | 780 | Hamburger, commercial (g) | 2413 |
| White cabbage, loose (g) | 235 | 1470 | Chicken in sandwich | 120 | 120 | Beef sausages (g) | 1048 |
| Iceberg lettuce, whole (g) | 795 | 1470 | Peanuts, roasted, unsalted | N/A | 780 | Ham (g) | 189 |
| Carrot, loose (g) | 753 | 2205 | ***Milk, yoghurt, cheese and alternatives*** | | | Potato crisps, pp (g) | 518 |
| Pumpkin (g) | 240 | 2205 | Cheddar cheese, full fat (g) | 624 | 704 | Potato chips, hot, commercial (g) | 670 |
| Four bean mix, canned (g) | 74 | 1005 | Cheddar cheese, reduced fat (g) | 44 | 516 | Ice cream (g) | 1830 |
| Diced tomatoes, canned in tomato juice (g) | 234 | 1638 | Milk, full fat (g) | 5961 | 6438 | White sugar (g) | 564 |
| Onion, brown, loose (g) | 84 | 1638 | Milk, reduced fat (g) | 2929 | 12000 | Salad dressing (ml) | 277 |
| Tomatoes, loose (g) | 488 | 1638 | Yoghurt, full fat, plain (g) | 204 | 2576 | Tomato sauce (ml) | 569 |
| Frozen mixed vegetables, pp (g) | 1184 | 1638 | Yoghurt, reduced fat, flavoured (vanilla) (g) | 676 | 5100 | Chicken soup, canned (g) | 1340 |
| Frozen peas, pp (g) | 273 | 1638 | Flavoured milk (ml) | 2416 | N/A | Orange juice (ml) | 3027 |
| Baked beans, canned (g) | 369 | 1005 | ***Unsaturated oils and spreads*** |  |  | Fish fillet crumbed, pp (g) | 302 |
| Salad vegetables in sandwich | 120 | 120 | Canola margarine (g) | 170 | 412 | Instant noodles, wheat based (g) | 381 |
| Vegetables in tinned meat and vegetable casserole (g) | 646 | N/A | Sunflower oil (ml) | 7 | 291 | ** The recommended diet does not contain discretionary choices.* | |
|  |  |  | Olive oil (ml) | 7 | 291 |  |  |
| *pp = pre-packaged* |  |  |  |  |  |  |  |
