# Supplementary material for: Affordability of current, and healthy, more equitable, sustainable diets by area of socioeconomic disadvantage and remoteness in Queensland: insights into food choice
Source: Int J Equity Health. 2021 Jun 30;20:153. doi: 10.1186/s12939-021-01481-8 (PMC8243618; doi:10.1186/s12939-021-01481-8)
Supplement: Supplementary file 2 — Additional file 2. Calculations of indicative low disposable household incomes. [file 12939_2021_1481_MOESM2_ESM.docx]

| **Income source** | **Indicative low disposable household income (in 2019)** | **Indicative low disposable household income, including government supplements due to the SARS-CoV-2 pandemic (in 2020)** |
| --- | --- | --- |
| Paid employment - adult male | $1481.24 | $1481.24 |
| Paid employment - adult female | $233.88 | $233.88 |
| JobSeeker | N/A | $324.05 |
| Family Tax Benefit A fortnightly payment | $428.40 | $428.40 |
| Family Tax Benefit A annual supplement | $57.84 ($751.90 per child per year) | $58.96 ($766.50 per child per year) |
| Family Tax Benefit B fortnightly payment | $107.66 | $74.06 |
| Family Tax Benefit B annual supplement | $14.04 ($365 per family per year) | $14.32 ($372.3 per family per year) |
| Total Clean Energy Supplement | N/A | $7.90 |
| Rent Assistance | $161.14 | $164.08 |
| Economic Support Payment | N/A | $125 (One off $750 payment in April 2020) |
| Coronavirus Supplement | N/A | $550.00 (from April 27 – September 24, 2020) |
| Income tax payable (including tax offsets & Medicare levy if applicable) | -$125.87 | -$125.87 |
| **Total fortnightly income** | **$2358.33** | **$3336.02** |
