# Supplementary material for: Affordability of current, and healthy, more equitable, sustainable diets by area of socioeconomic disadvantage and remoteness in Queensland: insights into food choice
Source: Int J Equity Health. 2021 Jun 30;20:153. doi: 10.1186/s12939-021-01481-8 (PMC8243618; doi:10.1186/s12939-021-01481-8)
Supplement: Supplementary file 3 — Additional file 3. Healthy Diets ASAP Survey Form. [file 12939_2021_1481_MOESM3_ESM.docx]

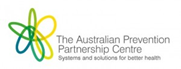

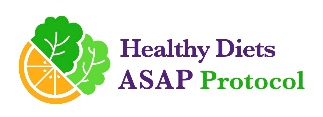

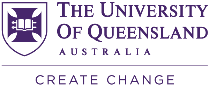


Date: ___________

Store name _____________________________________________

Store Location: __________________________________________

Collector:___________________________

**Price Collection Protocol**

1. Record the *usual price of an item*, i.e. do not collect the sale/special price unless it is the only price available (if so, note in comment column)
2. *Look for the specified brand and specified size for each food item, and record the price*

- *If the specified brand is not available*: Choose the cheapest brand (non-generic) available in the specified size. Note this brand in the “Your brand” column
- *If the specified size is not available*: Choose the nearest larger size in the specified brand. If a larger size is not available, choose the nearest smaller size. Note this size in the “Your size” column.
- *If both the specified brand and specified size are not available*: Choose the cheapest in the nearest larger size of another brand (non-generic). If a larger size is not available, choose the nearest smaller size.
- *If multiple brands are specified,* record the price of the cheapest one and note brand in the “Your brand” column
- *If the item is only available in a generic form* (e.g. Home Brand, Coles, Woolworths Select, Black and Gold) choose the *most expensive generic* item in the specified size. If the specified size is not available, choose the nearest larger size. If a larger size is not available, choose the nearest smaller size. Note the generic name in the “Your brand” and the size in the “Your size” columns.

1. *Loose produce*: choose the usual cheapest price per kg of the variety not on special. If the only variety available is on special, record the special price and note in comments column.
2. *Peanuts*: choose the branded packet size closest to 250g. If packaged, roasted, unsalted peanuts are not available, record the price of the loose ‘bulk – scoop & weight’ roasted, unsalted peanuts per 100g.
3. *Check all data are recorded as above before leaving store.*

For more information contact Prof Amanda Lee, School of Public Health, The University of Queensland e: [Amanda.Lee@uq.edu.au](mailto:Amanda.Lee@uq.edu.au) m: 0412975197


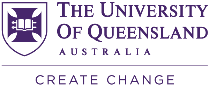

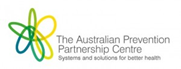

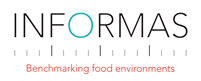

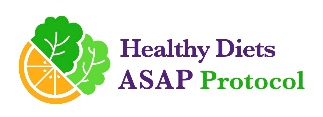


**Healthy Diets ASAP (Australian Standardised Affordability and Price) Survey Form**

| **Food** | **Specific brand** | **Your brand** | **Specific size** | **Your size** | **Your cost** | **Comments** |
| --- | --- | --- | --- | --- | --- | --- |
| Fresh Fruit |  |  |  |  |  |  |
| Apples, red, loose |  |  | per kg |  |  |  |
| Bananas, cavendish, loose |  |  | per kg |  |  |  |
| Orange, loose |  |  | per kg |  |  |  |
| Fresh Vegetables |  |  |  |  |  |  |
| White potato, loose, brushed/washed |  |  | per kg |  |  |  |
| Broccoli, loose |  |  | per kg |  |  |  |
| Cabbage, white, ½ cabbage (1/2=1.5kg) (weigh if necessary) |  |  | 1.5kg |  |  |  |
| Lettuce, iceberg, whole (1=0.6kg) |  |  | 0.6kg |  |  |  |
| Carrot, loose |  |  | per kg |  |  |  |
| Pumpkin, ½ pumpkin (1/2 av. Jap=1.5kg, 1/2 av. Butternut=1kg) (weigh if necessary) |  |  | per kg |  |  |  |
| Brown onion, loose |  |  | per kg |  |  |  |
| Tomato, loose (not vine-ripened) |  |  | per kg |  |  |  |
| Tinned Foods |  |  |  |  |  |  |
| Tinned sweet corn, kernels, no added salt | Edgell |  | 420g |  |  |  |
| Tinned 4 bean mix | Edgell |  | 420g |  |  |  |
| Tinned diced/chopped tomatoes, in tomato juice | Ardmona |  | 400g |  |  |  |
| Fruit salad, canned in juice | Goulburn Valley |  | 700g |  |  |  |
| Tinned steak & vegetables | Harvest |  | 425g |  |  |  |
| Tinned baked beans, in tomato sauce | Heinz |  | 420g |  |  |  |
| Tinned chicken & vegetable soup, ready to eat | Campbell’s Country Ladle |  | 505g |  |  |  |
| Tuna, canned in vegetable oil, unflavoured (cheapest specified brand) | John West, Greenseas or Sirena |  | 185g |  |  |  |
| Pantry Foods |  |  |  |  |  |  |
| Wholemeal Bread | Tip Top Sunblest |  | 650g |  |  |  |
| White Bread | Tip Top Sunblest |  | 650g |  |  |  |
| Muffin, commercial, uniced | Supermarket |  | $/100g |  |  |  |
| Rolled oats, whole, Traditional (not quick oats) | Uncle Toby’s |  | 1kg |  |  |  |
| Cornflakes | Kellogg’s |  | 725g |  |  |  |
| Weet-bix | Sanitarium |  | 375g |  |  |  |
| Spaghetti (white) | San Remo |  | 500g |  |  |  |
| White rice, medium grain | SunRice |  | 1kg |  |  |  |
| 2 Minute noodles, chicken (cheapest specified brand) | Maggi or Fantastic |  | 70g |  |  |  |
| White Sugar | CSR |  | 2kg |  |  |  |


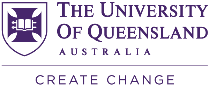

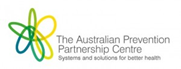

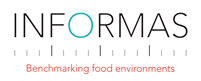

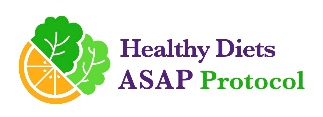


Store name __________________ Store Location: _______________ Date: ___________ Collector:_______

| **Food** | **Specific brand** | **Your brand** | **Specific size** | **Your size** | **Your cost** | **Comments** |
| --- | --- | --- | --- | --- | --- | --- |
| Cream-filled biscuit | Arnott’s Monte-Carlo |  | 250g |  |  |  |
| Chewy Choc Chip Muesli Bar | Uncle Toby’s |  | 6x30g (185g) |  |  |  |
| Water Crackers, plain | Arnott’s |  | 125g |  |  |  |
| Savoury flavoured biscuits | Arnott’s BBQ Shapes |  | 175g |  |  |  |
| Peanuts – roasted, unsalted peanuts | Cheapest branded |  | 250g |  |  |  |
| Mixed nuts, (incl. peanut), salted | Nobby’s |  | 375g |  |  |  |
| Mint confectionary | Allen’s Minties |  | 150g |  |  |  |
| Dairy milk chocolate, block | Cadbury |  | 200g |  |  |  |
| Chips/crisps, original, salted | Smith’s or Thins |  | 170g |  |  |  |
| French Dressing, regular fat | Praise |  | 330mL |  |  |  |
| Tomato sauce, regular (not ketchup) | Heinz Big Red or Masterfoods |  | 500mL |  |  |  |
| Sunflower oil | Crisco |  | 750mL |  |  |  |
| Olive oil, Traditional (not extra virgin) | Moro |  | 1 Litre |  |  |  |
| Meats |  |  |  |  |  |  |
| Lean beef mince (not heart smart) | Pre-pack(not vacuum) |  | per kg |  |  |  |
| Lamb loin chops | Pre-pack |  | per kg |  |  |  |
| Beef rump steak | Pre-pack |  | per kg |  |  |  |
| Beef Sausages, pre-pack | Supermarket |  | per kg |  |  |  |
| Refrigerated Items |  |  |  |  |  |  |
| Cheddar cheese, regular fat | Coon |  | 250g |  |  |  |
| Cheddar cheese, reduced fat | Coon |  | 250g |  |  |  |
| Butter, original, salted (foil pack) | Western Star |  | 250g |  |  |  |
| Canola Margarine, regular fat | MeadowLea |  | 500g |  |  |  |
| Full cream milk, fresh | Paul’s or Dairy Farmers |  | 2L |  |  |  |
| Reduced fat milk, fresh (not skim) | Paul’s Trim or Dairy Farmers Lite |  | 2L |  |  |  |
| Chocolate Milk, regular fat | Breaka, Big M,Oak or Paul’s |  | 600mL |  |  |  |
| Plain Yoghurt, natural, Greek, regular fat (~4% fat) | Jalna |  | 1kg |  |  |  |
| Yoghurt, vanilla/flavoured, reduced fat (~1% fat) | Jalna |  | 1kg |  |  |  |
| Leg Ham, pre-pack | Don’s |  | 250g |  |  |  |
| Eggs, dozen, Free Range | Sunnyqueen Farms |  | 700g |  |  |  |

| **Food** | **Specific brand** | **Your brand** | **Specific size** | **Your size** |  | **Your cost** | **Comments** |
| --- | --- | --- | --- | --- | --- | --- | --- |
| Drinks |  |  |  |  |  |  |  |
| Bottled water, still | Mt Franklin |  | 600mL |  |  |  |  |
| Soft drink, Cola | Coca Cola |  | 1.25L |  |  |  |  |
| Diet soft drink, Cola | Coca Cola |  | 1.25L |  |  |  |  |
| Orange Juice, Australian Grown (Fresh, chilled) | Berri |  | 2L |  |  |  |  |
| Frozen Foods |  |  |  |  |  |  |  |
| Frozen mixed vegetables (cheapest specified brand) | Heinz, Birdseye or McCain |  | 500g |  |  |  |  |
| Frozen peas (cheapest specified brand) | Edgell, Birdseye or McCain |  | 500g |  |  |  |  |
| Beef lasagne, frozen | McCain |  | 400g |  |  |  |  |
| White crumbed fish fillet, frozen | Birds Eye |  | 425g |  |  |  |  |
| Vanilla Ice cream, regular fat | Nestle Peters Original |  | 2L |  |  |  |  |
| Other Items | |  |  |  |  |  |  |
| Whole Barbeque Chicken, cooked - Large/ Family | Supermarket |  | Per unit ~1.5kg |  |  |  |  |
| Pre-made Chicken & Salad Sandwich (wholemeal) (1 sandwich = ~220g) | Supermarket or, if unavailable, at closest garage/service station |  | 2sl bread + filling  (triangle pre-pack) |  |  |  |  |

**
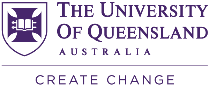
**
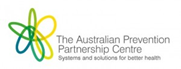

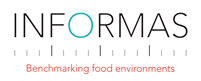

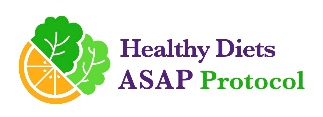

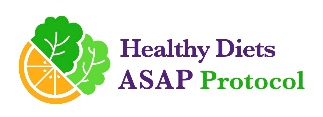
**
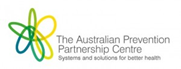
**

Items from other stores:

| **Food** | **Store** | **Your store** | **Specific size** | **Your size** | **Your cost** | **Comments** |
| --- | --- | --- | --- | --- | --- | --- |
| Cooked hot potato chips | Independent Fish & Chip shop |  | ~110g  1 serve |  |  |  |
| Beef hamburger (Big Mac) | McDonald’s |  | 1 burger |  |  |  |
| Beef Pie, single serve, full pastry | Independent Bakery |  | ~250g  1 pie |  |  |  |
| Supreme Pizza, thin base (1 pizza=0.55kg) | Pizza Hut |  | 1 large pizza |  |  |  |

Liquor Store Name: _____________________

| **Food** | **Specific brand** | **Your brand** | **Specific size** | **Your size** | **Your cost** | **Comments** |
| --- | --- | --- | --- | --- | --- | --- |
| Beer | VB |  | 6 x 375mL |  |  |  |
| Sparkling white wine | Yellow |  | 750mL |  |  |  |
| Whisky | Johnny Walker Red Label |  | 700mL |  |  |  |
| Red wine | Penfolds Koonungara Hill Shiraz |  | 750mL |  |  |  |
