# Supplementary material for: Affordability of current, and healthy, more equitable, sustainable diets by area of socioeconomic disadvantage and remoteness in Queensland: insights into food choice
Source: Int J Equity Health. 2021 Jun 30;20:153. doi: 10.1186/s12939-021-01481-8 (PMC8243618; doi:10.1186/s12939-021-01481-8)
Supplement: Supplementary file 4 — Additional file 4. Cost and affordability of the current and recommended diets in the sampled locations. [file 12939_2021_1481_MOESM4_ESM.docx]

| **SA2 area (deidentified)** | **Comm1** | | | | | | | **Comm2** | | | | | | | | **Comm3** | | | | | | | | | |
| --- | --- | --- | --- | --- | --- | --- | --- | --- | --- | --- | --- | --- | --- | --- | --- | --- | --- | --- | --- | --- | --- | --- | --- | --- | --- |
| **SEIFA SA2 quintile** | **1** | | | | | | | **1** | | | | | | | | **1** | | | | | | | | | |
| **ARIA+ category** | **Major cities** | | | | | | | **Major cities** | | | | | | | | **Major cities** | | | | | | | | | |
| **Total diet and food group costs** | | | | | | | | | | | | | | | | | | | | | | | | | |
| **Food/food groups** | **Current diet** | | | **Recommended diet** | | | | **Current diet** | | | **Recommended diet** | | | | | **Current diet** | | | | **Recommended diet** | | | | | |
|  | **Mean cost ± SD (A$)** | **Proportion of total cost (%)** | **Mean cost ± SD (A$)** | | **Proportion of total cost (%)** | | **Mean cost ± SD (A$)** | | **Proportion of total cost (%)** | **Mean cost ± SD (A$)** | | **Proportion of total cost (%)** | | | **Mean cost ± SD (A$)** | | | **Proportion of total cost (%)** | **Mean cost ± SD (A$)** | | | **Proportion of total cost (%)** | | |  |
| Water, bottled | 22.95 ± 4.01 | 3% | 22.95 ± 4.01 | | 4% | | 22.80 ± 3.82 | | 3% | 22.80 ± 3.82 | | 4% | | | 17.33 ± 4.44 | | | 2% | 17.33 ± 4.44 | | | 3% | | |  |
| Fruit | 49.75 ± 4.82 | 6% | 60.67 ± 12.47 | | 10% | | 54.53 ± 7.11 | | 7% | 68.81 ± 2.35 | | 11% | | | 56.23 ± 9.01 | | | 7% | 83.23 ± 20.09 | | | 13% | | |  |
| Vegetables (& legumes) | 43.08 ± 1.21 | 6% | 107.99 ± 3.63 | | 18% | | 42.34 ± 0.56 | | 6% | 109.35 ± 1.96 | | 18% | | | 44.43 ± 2.10 | | | 6% | 114.37 ± 5.42 | | | 19% | | |  |
| Grain (cereal) foods | 45.39 ± 5.78 | 6% | 112.32 ± 9.33 | | 18% | | 41.66 ± 1.58 | | 5% | 109.60 ± 3.06 | | 18% | | | 42.83 ± 1.08 | | | 6% | 108.29 ± 2.34 | | | 18% | | |  |
| Lean meats, poultry, fish, eggs, nuts, seeds & alternatives | 99.19 ± 14.91 | 13% | 188.22 ± 22.99 | | 31% | | 98.23 ± 4.37 | | 13% | 187.94 ± 10.88 | | 30% | | | 91.74 ± 12.87 | | | 12% | 176.15 ± 26.47 | | | 29% | | |  |
| Milk, yoghurt, cheese & alternatives | 48.40 ± 3.36 | 6% | 111.00 ± 6.20 | | 18% | | 49.40 ± 4.57 | | 6% | 110.85 ± 5.78 | | 18% | | | 47.81 ± 2.34 | | | 6% | 110.34 ± 5.07 | | | 18% | | |  |
| Unsaturated oils and spreads | 1.25 ± 0.03 | <1% | 8.07 ± 1.39 | | 1% | | 1.31 ± 0.09 | | <1% | 8.44 ± 0.73 | | 1% | | | 1.25 ± 0.01 | | | <1% | 8.08 ± 0.67 | | | 1% | | |  |
| Artificially sweetened beverages | 4.86 ± 0.11 | 1% | - | | - | | 5.80 ± 0.57 | | 1% | - | | - | | | 6.23 ± 1.08 | | | 1% | - | | | - | | |  |
| Sugar sweetened beverages | 29.31 ± 0.68 | 4% | - | | - | | 32.64 ± 3.35 | | 4% | - | | - | | | 32.96 ± 3.81 | | | 4% | - | | | - | | |  |
| Takeaway foods | 143.31 ± 0.00 | 19% | - | | - | | 140.76 ± 0.00 | | 18% | - | | - | | | 145.24 ± 0.00 | | | 19% | - | | | - | | |  |
| Alcoholic beverages | 99.79 ± 0.00 | 13% | - | | - | | 81.71 ± 0.00 | | 11% | - | | - | | | 99.79 ± 0.00 | | | 13% | - | | | - | | |  |
| All other discretionary choices | 181.56 ± 10.17 | 24% | - | | - | | 191.01 ± 23.07 | | 25% | - | | - | | | 184.90 ± 20.52 | | | 24% | - | | | - | | |  |
| **TOTAL diet** | **768.82 ± 13.12** | **100%** | **611.21 ± 21.07** | | **100%** | | **762.20 ± 36.38** | | **100%** | **617.79 ± 6.90** | | **100%** | | | **770.73 ± 22.01** | | | **100%** | **617.79 ± 6.47** | | | **100%** | | |  |
| Healthy foods and drinks | 314.85 ± 8.23 | 41% | **611.21 ± 21.07** | | **100%** | | 316.07 ± 10.71 | | 41% | 617.79 ± 6.90 | | 100% | | | 307.85 ± 4.54 | | | 40% | 617.79 ± 6.47 | | | 100% | | |  |
| Discretionary foods and drinks | 453.96 ± 9.86 | 59% | - | | - | | 446.13 ± 26.42 | | 59% | - | | - | | | 462.88 ± 24.25 | | | 60% | - | | | - | | |  |
| **Income and diet affordability** | | | | | | | | | | | | | | | | | | | | | | | | | |
| **Income categories** | **Income (A$)** | | | **Current diet affordability (% of income)** | | **Recommended diet affordability (% of income)** | | **Income (A$)** | | | **Current diet affordability (% of income)** | | **Recommended diet affordability (% of income)** | | | **Income (A$)** | | | | **Current diet affordability (% of income)** | | | **Recommended diet affordability (% of income)** | | |
| Median gross household income* | 1971.34 | | | 39% | | 31% | | 2272.66 | | | 34% | | 27% | | | 2340.57 | | | | 33% | | | 26% | | |
| Indicative low disposable household income | 2358.33 | | | 33% | | 26% | | 2358.33 | | | 32% | | 26% | | | 2358.33 | | | | 33% | | | 26% | | |
| Indicative low disposable household income including government supplements due to the SARS-CoV-2 pandemic | 3336.02 | | | 23% | | 18% | | 3336.02 | | | 23% | | 19% | | | 3336.02 | | | | 23% | | | 19% | | |
| **Mean of the median gross household income of all SA2 locations within the relevant classifications included* | | | | | | | | | | | | | |  | | |  | | | |  | | |  |  |

| **SA2 area (deidentified)** | **Comm4** | | | | | **Comm5** | | | | **Comm6** | | | |
| --- | --- | --- | --- | --- | --- | --- | --- | --- | --- | --- | --- | --- | --- |
| **SEIFA SA2 quintile** | **1** | | | | | **1** | | | | **1** | | | |
| **ARIA+ category** | **Outer regional** | | | | | **Outer regional** | | | | **Very remote** | | | |
| **Total diet and food group costs** | | | | | | | | | | | | | |
| **Food/food groups** | **Current diet** | | | **Recommended diet** | | **Current diet** | | **Recommended diet** | | **Current diet** | | **Recommended diet** | |
|  | **Mean cost ± SD (A$)** | **Proportion of total cost (%)** | **Mean cost ± SD (A$)** | | **Proportion of total cost (%)** | **Mean cost ± SD (A$)** | **Proportion of total cost (%)** | **Mean cost ± SD (A$)** | **Proportion of total cost (%)** | **Mean cost ± SD (A$)** | **Proportion of total cost (%)** | **Mean cost ± SD (A$)** | **Proportion of total cost (%)** |
| Water, bottled | 8.89 ± 0.64 | 1% | 8.89 ± 0.64 | | 1% | 14.68 ± 7.07 | 2% | 14.68 ± 7.07 | 2% | 8.83 ± 0.00 | 1% | 8.83 ± 0.00 | 1% |
| Fruit | 56.98 ± 0.03 | 8% | 79.07 ± 4.94 | | 12% | 56.52 ± 1.61 | 8% | 89.65 ± 2.01 | 15% | 64.87 ± 0.00 | 6% | 95.91 ± 0.00 | 11% |
| Vegetables (& legumes) | 41.45 ± 0.87 | 6% | 113.99 ± 0.37 | | 18% | 40.01 ± 0.22 | 5% | 105.04 ± 4.34 | 17% | 48.64 ± 0.00 | 4% | 118.47 ± 0.00 | 14% |
| Grain (cereal) foods | 41.75 ± 3.16 | 6% | 106.06 ± 3.42 | | 17% | 41.15 ± 0.75 | 5% | 98.90 ± 5.39 | 16% | 62.70 ± 0.00 | 6% | 152.38 ± 0.00 | 18% |
| Lean meats, poultry, fish, eggs, nuts, seeds & alternatives | 105.39 ± 0.44 | 15% | 209.27 ± 5.95 | | 33% | 97.12 ± 3.22 | 13% | 186.06 ± 6.14 | 30% | 134.30 ± 0.00 | 12% | 275.45 ± 0.00 | 32% |
| Milk, yoghurt, cheese & alternatives | 50.73 ± 0.78 | 7% | 111.88 ± 0.98 | | 18% | 49.15 ± 1.01 | 7% | 113.44 ± 3.20 | 18% | 76.11 ± 0.00 | 7% | 183.82 ± 0.00 | 22% |
| Unsaturated oils and spreads | 1.25 ± 0.00 | <1% | 8.24 ± 0.05 | | 1% | 1.05 ± 0.25 | <1% | 7.05 ± 0.83 | 1% | 1.75 ± 0.00 | <1% | 13.10 ± 0.00 | 2% |
| Artificially sweetened beverages | 6.02 ± 0.00 | 1% | - | | - | 5.16 ± 1.02 | 1% | - | - | 9.93 ± 0.00 | 1% | - | - |
| Sugar sweetened beverages | 30.27 ± 0.00 | 4% | - | | - | 25.95 ± 5.13 | 3% | - | - | 51.80 ± 0.00 | 5% | - | - |
| Takeaway foods | 86.69 ± 0.00 | 12% | - | | - | 159.38 ± 0.00 | 21% | - | - | 241.59 ± 0.00 | 22% | - | - |
| Alcoholic beverages | 102.97 ± 0.00 | 15% | - | | - | 97.99 ± 0.00 | 13% | - | - | 79.46 ± 0.00 | 7% | - | - |
| All other discretionary choices | 174.26 ± 11.8 | 25% | - | | - | 163.57 ± 2.48 | 22% | - | - | 316.52 ± 0.00 | 29% | - | - |
| **TOTAL diet** | **706.66 ± 14.43** | **100%** | **637.39 ± 13.65** | | **100%** | **751.73 ± 18.06** | **100%** | **614.82 ± 5.48** | **100%** | **1096.47 ± 0.00** | **100%** | **847.96 ± 0.00** | **100%** |
| Healthy foods and drinks | 312.48 ± 2.63 | 44% | 637.39 ± 13.65 | | 100% | 304.84 ± 10.50 | 41% | 614.82 ± 5.48 | 100% | 407.11 ± 0.00 | 37% | 847.96 ± 0.00 | 100% |
| Discretionary foods and drinks | 394.18 ± 11.80 | 56% | - | | - | 446.89 ± 7.60 | 59% | - | - | 689.36 ± 0.00 | 63% | - | - |
| **Income and diet affordability** | | | | | | | | | | | | | |
| **Income categories** | **Income (A$)** | | | **Current diet affordability (% of income)** | **Recommended diet affordability (% of income)** | **Income (A$)** | | **Current diet affordability (% of income)** | **Recommended diet affordability (% of income)** | **Income (A$)** | | **Current diet affordability (% of income)** | **Recommended diet affordability (% of income)** |
| Median gross household income* | 2090.17 | | | 38% | 31% | 2166.56 | | 35% | 28% | 2105.02 | | 52% | 40% |
| Indicative low disposable household income | 2358.33 | | | 34% | 28% | 2358.33 | | 32% | 26% | 2358.33 | | 46% | 36% |
| Indicative low disposable household income including government supplements due to the SARS-CoV-2 pandemic | 3336.02 | | | 24% | 20% | 3336.02 | | 23% | 18% | 3336.02 | | 33% | 25% |
| **Mean of the median gross household income of all SA2 locations within the relevant classifications included* | | | | | | | | |  |  | |  |  |
| **SA2 area (deidentified)** | **Comm7** | | | | | **Comm8** | | | | **Comm9** | | | |
| **SEIFA SA2 quintile** | **3** | | | | | **3** | | | | **3** | | | |
| **ARIA+ category** | **Major cities** | | | | | **Major cities** | | | | **Major cities** | | | |
| **Total diet and food group costs** | | | | | | | | | | | | | |
| **Food/food groups** | **Current diet** | | | **Recommended diet** | | **Current diet** | | **Recommended diet** | | **Current diet** | | **Recommended diet** | |
|  | **Mean cost ± SD (A$)** | **Proportion of total cost (%)** | **Mean cost ± SD (A$)** | | **Proportion of total cost (%)** | **Mean cost ± SD (A$)** | **Proportion of total cost (%)** | **Mean cost ± SD (A$)** | **Proportion of total cost (%)** | **Mean cost ± SD (A$)** | **Proportion of total cost (%)** | **Mean cost ± SD (A$)** | **Proportion of total cost (%)** |
| Water, bottled | 20.83 ± 1.62 | 3% | 20.83 ± 1.62 | | 3% | 20.15 ± 1.85 | 3% | 20.15 ± 1.85 | 3% | 21.19 ± 1.92 | 3% | 21.19 ± 1.92 | 4% |
| Fruit | 50.68 ± 2.36 | 7% | 76.88 ± 3.68 | | 12% | 58.09 ± 8.65 | 7% | 82.85 ± 11.00 | 13% | 51.97 ± 6.09 | 7% | 73.66 ± 12.58 | 13% |
| Vegetables (& legumes) | 40.63 ± 1.24 | 5% | 102.39 ± 3.61 | | 17% | 45.52 ± 2.32 | 6% | 115.21 ± 6.08 | 18% | 41.48 ± 1.93 | 5% | 104.45 ± 6.74 | 18% |
| Grain (cereal) foods | 42.44 ± 0.79 | 6% | 107.24 ± 1.35 | | 17% | 43.50 ± 0.65 | 5% | 109.68 ± 1.98 | 17% | 43.77 ± 2.66 | 6% | 108.64 ± 3.32 | 18% |
| Lean meats, poultry, fish, eggs, nuts, seeds & alternatives | 99.19 ± 6.53 | 13% | 192.30 ± 13.77 | | 31% | 99.78 ± 0.75 | 13% | 193.55 ± 2.89 | 30% | 91.58 ± 5.45 | 12% | 172.28 ± 15.50 | 29% |
| Milk, yoghurt, cheese & alternatives | 48.28 ± 2.44 | 6% | 110.88 ± 1.10 | | 18% | 49.63 ± 4.10 | 6% | 113.72 ± 6.11 | 18% | 41.29 ± 3.20 | 5% | 99.56 ± 1.19 | 17% |
| Unsaturated oils and spreads | 1.23 ± 0.03 | <1% | 8.55 ± 0.88 | | 1% | 1.26 ± 0.03 | <1% | 8.81 ± 1.27 | 1% | 1.26 ± 0.02 | <1% | 8.57 ± 0.93 | 1% |
| Artificially sweetened beverages | 6.02 ± 0.00 | 1% | - | | - | 5.59 ± 0.42 | 1% | - | - | 6.01 ± 1.39 | 1% | - | - |
| Sugar sweetened beverages | 29.79 ± 0.68 | 4% | - | | - | 29.79 ± 0.68 | 4% | - | - | 33.54 ± 4.62 | 4% | - | - |
| Takeaway foods | 154.39 ± 0.00 | 20% | - | | - | 146.51 ± 0.00 | 18% | - | - | 164.25 ± 0.00 | 21% | - | - |
| Alcoholic beverages | 99.79 ± 0.00 | 13% | - | | - | 99.79 ± 0.00 | 13% | - | - | 95.3 ± 0.00 | 12% | - | - |
| All other discretionary choices | 170.57 ± 3.51 | 22% | - | | - | 193.48 ± 29.38 | 24% | - | - | 173.48 ± 1.97 | 23% | - | - |
| **TOTAL diet** | **763.83 ± 4.04** | **100%** | **619.08 ± 4.55** | | **100%** | **793.08 ± 42.98** | **100%** | **643.97 ± 26.69** | **100%** | **765.11 ± 16.60** | **100%** | **588.34 ± 14.55** | **100%** |
| Healthy foods and drinks | 309.30 ± 0.87 | 40% | 619.08 ± 4.55 | | 100% | 323.52 ± 14.28 | 41% | 643.97 ± 26.69 | 100% | 298.55 ± 10.90 | 39% | 588.34 ± 14.55 | 100% |
| Discretionary foods and drinks | 454.53 ± 4.15 | 60% | - | | - | 469.57 ± 28.7 | 59% | - | - | 466.56 ± 6.40 | 61% | - | - |
| **Income and diet affordability** | | | | | | | | | | | | | |
| **Income categories** | **Income (A$)** | | | **Current diet affordability (% of income)** | **Recommended diet affordability (% of income)** | **Income (A$)** | | **Current diet affordability (% of income)** | **Recommended diet affordability (% of income)** | **Income (A$)** | | **Current diet affordability (% of income)** | **Recommended diet affordability (% of income)** |
| Median gross household income | 2822.26 | | | 27% | 22% | 3388.83 | | 23% | 19% | 2958.07 | | 26% | 20% |
| Indicative low disposable household income | 2358.33 | | | 32% | 26% | 2358.33 | | 34% | 27% | 2358.33 | | 32% | 25% |
| Indicative low disposable household income including government supplements due to the SARS-CoV-2 pandemic | 3336.02 | | | 23% | 19% | 3336.02 | | 24% | 19% | 3336.02 | | 23% | 18% |
| **Mean of the median gross household income of all SA2 locations within the relevant classifications included* | | | | | | | | |  |  | |  |  |
| **SA2 area (deidentified)** | **Comm10** | | | | | **Comm11** | | | | **Comm12** | | | |
| **SEIFA SA2 quintile** | **3** | | | | | **3** | | | | **3** | | | |
| **ARIA+ category** | **Major cities** | | | | | **Outer regional** | | | | **Outer regional** | | | |
| **Total diet and food group costs** | | | | | | | | | | | | | |
| **Food/food groups** | **Current diet** | | | **Recommended diet** | | **Current diet** | | **Recommended diet** | | **Current diet** | | **Recommended diet** | |
|  | **Mean cost ± SD (A$)** | **Proportion of total cost (%)** | **Mean cost ± SD (A$)** | | **Proportion of total cost (%)** | **Mean cost ± SD (A$)** | **Proportion of total cost (%)** | **Mean cost ± SD (A$)** | **Proportion of total cost (%)** | **Mean cost ± SD (A$)** | **Proportion of total cost (%)** | **Mean cost ± SD (A$)** | **Proportion of total cost (%)** |
| Water, bottled | 18.98 ± 2.36 | 3% | 18.98 ± 2.36 | | 3% | 16.77 ± 11.60 | 2% | 16.77 ± 11.60 | 3% | 17.46 ± 7.12 | 2% | 17.46 ± 7.12 | 3% |
| Fruit | 50.19 ± 5.07 | 7% | 67.52 ± 8.55 | | 11% | 58.01 ± 3.92 | 7% | 82.93 ± 1.88 | 13% | 52.65 ± 2.88 | 7% | 75.80 ± 4.86 | 13% |
| Vegetables (& legumes) | 44.66 ± 0.97 | 6% | 113.42 ± 3.94 | | 19% | 47.36 ± 0.94 | 5% | 118.94 ± 1.76 | 18% | 41.01 ± 1.43 | 5% | 101.17 ± 4.27 | 17% |
| Grain (cereal) foods | 44.26 ± 2.91 | 6% | 110.55 ± 5.34 | | 18% | 51.14 ± 5.85 | 6% | 118.91 ± 11.61 | 18% | 44.09 ± 0.60 | 6% | 107.27 ± 3.57 | 18% |
| Lean meats, poultry, fish, eggs, nuts, seeds & alternatives | 96.76 ± 4.42 | 13% | 181.94 ± 9.03 | | 30% | 103.46 ± 11.84 | 12% | 194.07 ± 21.63 | 29% | 91.26 ± 5.69 | 12% | 180.38 ± 10.92 | 30% |
| Milk, yoghurt, cheese & alternatives | 46.03 ± 0.60 | 6% | 107.10 ± 0.90 | | 18% | 50.59 ± 4.21 | 6% | 119.26 ± 7.95 | 18% | 45.18 ± 1.3 | 6% | 107.13 ± 6.05 | 18% |
| Unsaturated oils and spreads | 1.19 ± 0.06 | <1% | 7.89 ± 0.84 | | 1% | 1.35 ± 0.37 | <1% | 9.01 ± 3.01 | 1% | 1.07 ± 0.21 | <1% | 7.94 ± 1.48 | 1% |
| Artificially sweetened beverages | 4.94 ± 0.11 | 1% | - | | - | 6.64 ± 0.44 | 1% | - | - | 4.73 ± 1.19 | 1% | - | - |
| Sugar sweetened beverages | 29.79 ± 0.68 | 4% | - | | - | 33.35 ± 2.22 | 4% | - | - | 25.47 ± 6.79 | 3% | - | - |
| Takeaway foods | 145.36 ± 0.00 | 19% | - | | - | 208.42 ± 0.00 | 24% | - | - | 153.09 ± 0.00 | 20% | - | - |
| Alcoholic beverages | 99.79 ± 0.00 | 13% | - | | - | 96.41 ± 0.00 | 11% | - | - | 99.79 ± 0.00 | 13% | - | - |
| All other discretionary choices | 174.62 ± 6.17 | 23% | - | | - | 209.49 ± 37.96 | 24% | - | - | 174.95 ± 11.63 | 23% | - | - |
| **TOTAL diet** | **756.56 ± 16.64** | **100%** | **607.39 ± 25.46** | | **100%** | **882.97 ± 69.43** | **100%** | **659.90 ± 46.69** | **100%** | **750.74 ± 7.48** | **100%** | **597.17 ± 15.07** | **100%** |
| Healthy foods and drinks | 307.00 ± 11.12 | 41% | 607.39 ± 25.46 | | 100% | 335.31 ± 31.01 | 38% | 659.90 ± 46.69 | 100% | 297.45 ± 9.06 | 40% | 597.17 ± 15.07 | 100% |
| Discretionary foods and drinks | 449.56 ± 5.52 | 59% | - | | - | 547.67 ± 40.11 | 62% | - | - | 453.29 ± 4.85 | 60% | - | - |
| **Income and diet affordability** | | | | | | | | | | | | | |
| **Income categories** | **Income (A$)** | | | **Current diet affordability (% of income)** | **Recommended diet affordability (% of income)** | **Income (A$)** | | **Current diet affordability (% of income)** | **Recommended diet affordability (% of income)** | **Income (A$)** | | **Current diet affordability (% of income)** | **Recommended diet affordability (% of income)** |
| Median gross household income* | 3172.39 | | | 18% | 19% | 2236.59 | | 39% | 30% | 3997.85 | | 19% | 15% |
| Indicative low disposable household income | 2358.33 | | | 32% | 26% | 2358.33 | | 37% | 28% | 2358.33 | | 32% | 25% |
| Indicative low disposable household income including government supplements due to the SARS-CoV-2 pandemic | 3336.02 | | | 23% | 18% | 3336.02 | | 26% | 20% | 3336.02 | | 23% | 18% |
| **Mean of the median gross household income of all SA2 locations within the relevant classifications included* | | | | | | | | |  |  | |  |  |

| **SA2 area (deidentified)** | **Comm13** | | | | | **Comm14** | | | | **Comm15** | | | |
| --- | --- | --- | --- | --- | --- | --- | --- | --- | --- | --- | --- | --- | --- |
| **SEIFA SA2 quintile** | **3** | | | | | **5** | | | | **5** | | | |
| **ARIA+ category** | **Very remote** | | | | | **Major cities** | | | | **Major cities** | | | |
| **Total diet and food group costs** | | | | | | | | | | | | | |
| **Food/food groups** | **Current diet** | | | **Recommended diet** | | **Current diet** | | **Recommended diet** | | **Current diet** | | **Recommended diet** | |
|  | **Mean cost ± SD (A$)** | **Proportion of total cost (%)** | **Mean cost ± SD (A$)** | | **Proportion of total cost (%)** | **Mean cost ± SD (A$)** | **Proportion of total cost (%)** | **Mean cost ± SD (A$)** | **Proportion of total cost (%)** | **Mean cost ± SD (A$)** | **Proportion of total cost (%)** | **Mean cost ± SD (A$)** | **Proportion of total cost (%)** |
| Water, bottled | 18.09 ± 5.74 | 2% | 18.09 ± 5.74 | | 2% | 18.98 ± 1.30 | 2% | 18.98 ± 1.30 | 3% | 20.30 ± 1.44 | 3% | 20.30 ± 1.44 | 3% |
| Fruit | 73.54 ± 1.82 | 7% | 113.15 ± 8.86 | | 15% | 62.32 ± 15.37 | 8% | 83.17 ± 18.38 | 12% | 49.80 ± 3.33 | 7% | 67.60 ± 1.62 | 11% |
| Vegetables (& legumes) | 52.99 ± 1.14 | 5% | 134.41 ± 0.06 | | 17% | 47.35 ± 4.86 | 6% | 121.05 ± 11.92 | 18% | 42.94 ± 1.63 | 6% | 109.58 ± 5.65 | 18% |
| Grain (cereal) foods | 52.48 ± 2.84 | 5% | 134.20 ± 0.66 | | 17% | 46.40 ± 6.27 | 6% | 108.36 ± 11.67 | 16% | 44.33 ± 4.24 | 6% | 109.36 ± 5.56 | 18% |
| Lean meats, poultry, fish, eggs, nuts, seeds & alternatives | 117.37 ± 0.83 | 11% | 230.96 ± 2.21 | | 30% | 102.12 ± 7.13 | 13% | 199.54 ± 14.07 | 30% | 92.80 ± 7.26 | 12% | 179.36 ± 6.21 | 30% |
| Milk, yoghurt, cheese & alternatives | 58.16 ± 2.46 | 6% | 136.43 ± 9.53 | | 18% | 52.52 ± 8.71 | 7% | 133.51 ± 36.24 | 20% | 48.88 ± 3.94 | 6% | 110.12 ± 5.36 | 18% |
| Unsaturated oils and spreads | 1.55 ± 0.08 | <1% | 11.16 ± 0.04 | | 1% | 1.35 ± 0.15 | <1% | 9.22 ± 1.84 | 1% | 1.25 ± 0.02 | <1% | 8.23 ± 0.87 | 1% |
| Artificially sweetened beverages | 7.77 ± 1.02 | 1% | - | | - | 5.89 ± 1.23 | 1% | - | - | 5.69 ± 0.47 | 1% | - | - |
| Sugar sweetened beverages | 39.06 ± 5.14 | 4% | - | | - | 32.96 ± 3.81 | 4% | - | - | 30.27 ± 0.00 | 4% | - | - |
| Takeaway foods | 209.55 ± 10.79 | 20% | - | | - | 147.47 ± 0.00 | 18% | - | - | 158.58 ± 0.00 | 21% | - | - |
| Alcoholic beverages | 99.78 ± 0.00 | 10% | - | | - | 88.11 ± 0.00 | 11% | - | - | 99.79 ± 0.00 | 13% | - | - |
| All other discretionary choices | 292.31 ± 56.66 | 29% | - | | - | 199.13 ± 38.46 | 25% | - | - | 170.81 ± 2.86 | 22% | - | - |
| **TOTAL diet** | **1022.67 ± 48.89** | **100%** | **778.40 ± 15.51** | | **100%** | **804.61 ± 81.97** | **100%** | **673.82 ± 87.34** | **100%** | **765.44 ± 9.33** | **100%** | **604.55 ± 13.17** | **100%** |
| Healthy foods and drinks | 381.97 ± 2.13 | 37% | 778.40 ± 15.51 | | 100% | 336.93 ± 39.82 | 42% | 673.82 ± 87.34 | 100% | 305.99 ± 6.89 | 40% | 604.55 ± 13.17 | 100% |
| Discretionary foods and drinks | 640.70 ± 51.01 | 63% | - | | - | 467.67 ± 42.26 | 58% | - | - | 459.45 ± 2.86 | 60% | - | - |
| **Income and diet affordability** | | | | | | | | | | | | | |
| **Income categories** | **Income (A$)** | | | **Current diet affordability (% of income)** | **Recommended diet affordability (% of income)** | **Income (A$)** | | **Current diet affordability (% of income)** | **Recommended diet affordability (% of income)** | **Income (A$)** | | **Current diet affordability (% of income)** | **Recommended diet affordability (% of income)** |
| Median gross household income* | 2707.67 | | | 38% | 29% | 4110.31 | | 20% | 16% | 4263.10 | | 18% | 14% |
| Indicative low disposable household income | 2358.33 | | | 43% | 33% | 2358.33 | | 34% | 29% | 2358.33 | | 32% | 26% |
| Indicative low disposable household income including government supplements due to the SARS-CoV-2 pandemic | 3336.02 | | | 31% | 23% | 3336.02 | | 24% | 20% | 3336.02 | | 23% | 18% |
| **Mean of the median gross household income of all SA2 locations within the relevant classifications included* | | | | | | | | |  |  | |  |  |

| **SA2 area (deidentified)** | **Comm16** | | | | **Comm17** | | | | | |
| --- | --- | --- | --- | --- | --- | --- | --- | --- | --- | --- |
| **SEIFA SA2 quintile** | **5** | | | | **5** | | | | | |
| **ARIA+ category** | **Major cities** | | | | **Outer regional** | | | | | |
| **Total diet and food group costs** | | | | | | | | | | |
| **Food/food groups** | **Current diet** | | **Recommended diet** | | **Current diet** | | **Recommended diet** | | | |
|  | **Mean cost ± SD (A$)** | **Proportion of total cost (%)** | **Mean cost ± SD (A$)** | **Proportion of total cost (%)** | **Mean cost ± SD (A$)** | **Proportion of total cost (%)** | **Mean cost ± SD (A$)** | | **Proportion of total cost (%)** | |
| Water, bottled | 20.01 ± 1.50 | 3% | 20.01 ± 1.50 | 3% | 20.08 ± 0.54 | 3% | 20.08 ± 0.54 | | 3% | |
| Fruit | 50.28 ± 5.24 | 7% | 63.72 ± 4.53 | 11% | 56.52 ± 1.61 | 7% | 89.65 ± 2.01 | | 14% | |
| Vegetables (& legumes) | 43.51 ± 1.97 | 6% | 105.85 ± 6.35 | 17% | 36.02 ± 0.67 | 5% | 105.34 ± 4.37 | | 17% | |
| Grain (cereal) foods | 48.81 ± 7.64 | 6% | 115.84 ± 10.47 | 19% | 37.14 ± 0.75 | 5% | 100.06 ± 5.1 | | 16% | |
| Lean meats, poultry, fish, eggs, nuts, seeds & alternatives | 93.10 ± 3.73 | 12% | 173.89 ± 5.42 | 29% | 92.52 ± 4.54 | 12% | 190.57 ± 13.91 | | 30% | |
| Milk, yoghurt, cheese & alternatives | 47.10 ± 7.31 | 6% | 118.86 ± 23.35 | 20% | 49.15 ± 1.01 | 6% | 113.46 ± 3.22 | | 18% | |
| Unsaturated oils and spreads | 1.40 ± 0.23 | <1% | 8.33 ± 1.04 | 1% | 1.05 ± 0.25 | <1% | 7.05 ± 0.84 | | 1% | |
| Artificially sweetened beverages | 5.38 ± 0.51 | 1% | - | - | 5.16 ± 1.02 | 1% | - | | - | |
| Sugar sweetened beverages | 30.40 ± 0.18 | 4% | - | - | 25.95 ± 5.13 | 3% | - | | - | |
| Takeaway foods | 147.28 ± 0.00 | 19% | - | - | 174.44 ± 0.00 | 23% | - | | - | |
| Alcoholic beverages | 99.79 ± 0.00 | 13% | - | - | 101.53 ± 0.00 | 13% | - | | - | |
| All other discretionary choices | 184.62 ± 18.04 | 24% | - | - | 171.74 ± 2.88 | 22% | - | | - | |
| **TOTAL diet** | **771.67 ± 41.32** | **100%** | **606.48 ± 40.68** | **100%** | **771.30 ± 5.66** | **100%** | **626.21 ± 6.64** | | **100%** | |
| Healthy foods and drinks | 309.58 ± 23.48 | 40% | 606.48 ± 40.68 | 100% | 297.64 ± 4.05 | 39% | 626.21 ± 6.64 | | 100% | |
| Discretionary foods and drinks | 462.09 ± 18.22 | 60% | - | - | 473.66 ± 3.06 | 61% | - | | - | |
| **Income and diet affordability** | | | | | | | | | | |
| **Income categories** | **Income (A$)** | | **Current diet affordability (% of income)** | **Recommended diet affordability (% of income)** | **Income (A$)** | | **Current diet affordability (% of income)** | | **Recommended diet affordability (% of income)** | |
| Median gross household income* | 4581.40 | | 17% | 13% | 4011.64 | | 20% | | 16% | |
| Indicative low disposable household income | 2358.33 | | 33% | 26% | 2358.33 | | 33% | | 27% | |
| Indicative low disposable household income including government supplements due to the SARS-CoV-2 pandemic | 3336.02 | | 23% | 18% | 3336.02 | | 24% | | 19% | |
| **Mean of the median gross household income of all SA2 locations within the relevant classifications included* | | | | | | | |  | |  |
